# Identification of Crosstalk between Phosphoprotein Signaling Pathways in RAW 264.7 Macrophage Cells

**DOI:** 10.1371/journal.pcbi.1000654

**Published:** 2010-01-29

**Authors:** Shakti Gupta, Mano Ram Maurya, Shankar Subramaniam

**Affiliations:** 1 Department of Bioengineering, University of California, San Diego, La Jolla, California, United States of America; 2 Department of Chemistry, University of California, San Diego, La Jolla, California, United States of America; 3 Department of Biochemistry, University of California, San Diego, La Jolla, California, United States of America; 4 Cellular & Molecular Medicine, University of California, San Diego, La Jolla, California, United States of America; 5 Graduate Program in Bioinformatics, University of California, San Diego, La Jolla, California, United States of America; Massachusetts Institute of Technology, United States of America

## Abstract

Signaling pathways mediate the effect of external stimuli on gene expression in cells. The signaling proteins in these pathways interact with each other and their phosphorylation levels often serve as indicators for the activity of signaling pathways. Several signaling pathways have been identified in mammalian cells but the crosstalk between them is not well understood. Alliance for Cellular Signaling (AfCS) has measured time-course data in RAW 264.7 macrophage cells on important phosphoproteins, such as the mitogen-activated protein kinases (MAPKs) and signal transducer and activator of transcription (STATs), in single- and double-ligand stimulation experiments for 22 ligands. In the present work, we have used a data-driven approach to analyze the AfCS data to decipher the interactions and crosstalk between signaling pathways in stimulated macrophage cells. We have used dynamic mapping to develop a predictive model using a partial least squares approach. Significant interactions were selected through statistical hypothesis testing and were used to reconstruct the phosphoprotein signaling network. The proposed data-driven approach is able to identify most of the known signaling interactions such as protein kinase B (Akt) → glycogen synthase kinase 3α/β (GSKα/β) etc., and predicts potential novel interactions such as P38 → RSK and GSK → ezrin/radixin/moesin. We have also shown that the model has good predictive power for extrapolation. Our novel approach captures the temporal causality and directionality in intracellular signaling pathways. Further, case specific analysis of the phosphoproteins in the network has led us to propose hypothesis about inhibition (phosphorylation) of GSKα/β via P38.

## Author Summary

Cellular systems are dynamic in nature, perform various biological functions and can adapt to their environment through compositional and structural remodeling. This remodeling is initiated by the binding of external ligands to receptor proteins on the cell surface or other stimuli resulting in the activation of various signaling pathways. The activation of signaling pathways in turn results in altered gene-expression which leads to changes in the molecular composition both inside and outside the cell. Thus, a thorough study of the complex interconnectivity of signaling pathways using dynamic data is necessary to understand the biological function and environmental adaptation. Protein phosphorylation is a key event in the activation of signaling pathways. We have developed an advanced computational approach to decipher the connections between different signaling pathways using time-resolved phosphoprotein data provided by the Alliance for Cellular Signaling. Important signaling events such as activation of glycogen synthase kinase 3 by protein kinase B (Akt) are captured by our reconstructed network. Novel links as well as testable hypotheses are also generated by our analysis approach.

## Introduction

Cells regulate their function through a complex circuitry that involves myriad interacting networks from intracellular signaling and metabolic pathways to genetic regulatory pathways. Intracellular signaling is the first step in translating the environmental cue in regulating various processes e.g. cell growth, differentiation and apoptosis. Activation of proteins through phosphorylation is an important event in intracellular signaling and serves as a metric for the flux in the signaling pathway. Understanding of the regulation of protein phosphorylation is the key to identifying cellular mechanisms which interpret the environmental cues. The knowledge of the regulation and interaction of various phosphoproteins is sparse. The goal of this paper is to develop an approach for data-driven reconstruction of phosphoprotein signaling networks and to test them using the large-scale phosphoprotein data available from the Alliance for Cellular Signaling (AfCS).

Among many types of posttranslational modifications of proteins, protein phosphorylation is the most studied and has substantial impact on biological function [1],[2]. Phosphorylation occurs on serine, threonine or tyrosine residues. Phosphoproteins (PPs) are considered as markers of signaling pathways because the levels of phosphorylation generally indicate the level of signaling activity in the pathway. The gamut of cellular processes affected by phosphoproteins varies from signal transduction, gene-expression, post-translational modifications of other proteins, cell differentiation, and development to cell cycle control. For example, the extracellular signal-regulated kinase (ERK) and P38 pathways are involved in cellular differentiation/proliferation [3]. c-Jun N-terminal kinase pathway is involved in cytokine induced apoptosis (tumor necrosis factor (TNF) signaling). In a similar manner, the other signaling pathways mediate important processes such as inflammation, the most prominent one is the P50–P65 nuclear-factor kappa beta (NF-KB) pathway [4],[5] Some molecules in these pathways, such as P50–P65 NF-KB and P38 translocate to the nucleus and act as transcription factors or regulate gene-expression through other mechanisms. In short, the ability to measure phosphoproteins has equipped the biologists to study the role of cytosolic intracellular signaling in virtually all aspects of biological processes.

Many of the signaling pathways function as a cascade. In a cascade, subsequent proteins are activated via a previous (phosphorylated/activated) protein. For example, in the P38 MAPK pathway, the cascade is composed of MAP or ERK kinase kinase 4 (MEKK4) and TGF-beta activated kinase 1 (TAK1) (a MAPK kinase kinase or MAPKKK), MKK3 and MKK6 (a MAP kinase kinase or MAPKK), and p38 MAPK [3],[6]. This pathway is activated by stress signals. Upon activation of MAPKKK, it phosphorylates and activates MAPKK which in turn phosphorylates and activates P38 MAPK. The information flows downstream with time, thus these interactions are causal in nature and can only be captured by dynamic mapping. Some pathways are activated in a non-cascade manner. One example is the cAMP signaling pathway in which upon production of cAMP within the cell through activation of adenylate cyclase via G-protein G_sα_, cAMP activates protein kinase A (PKA) which in turn stimulates degradation of cAMP itself through phosphorylation of a phosphodiesterase (an example of a negative feedback) [7]. Even in such pathways, information flow is generally downstream with time, e.g. G_sα_ → adenylate cyclase → cAMP → PKA. The availability of the temporally resolved measurements of many phosphoproteins allows us to study signaling pathways and cross-talk between them.

The development of high-throughput technologies have made these studies of pathways possible by allowing distinct types of simultaneous quantitative measurements of the cellular components such as mRNA levels, protein phosphorylations and metabolites. While lack of large datasets is one limiting factor in detailed and quantitative studies of regulation of signaling and metabolic pathways, such studies have also been impeded by the unavailability of suitable mathematical approaches to integrate diverse types of data and knowledge. Further, the complexity of intracellular signaling arising from feedback and feed-forward loops and cross-talk between different signaling pathways has exacerbated the problems associated with developing reliable mathematical approaches [8],[9]. This complexity is manifested by the presence of multiple time-scales ranging from few seconds to several hours across various biochemical processes. Data measured at accordingly appropriate time intervals are required to reconstruct causal networks for such processes. The differences in the time-scales and lack of knowledge about time-lags in various processes make it difficult to decipher their interactions. However, systems biology approaches open avenues to decipher the interactions between the components and aid partial reconstruction of the underlying cellular network.

Computational systems biology has seen tremendous advances during this decade. In the past few years, research in computational systems biology has moved beyond simple clustering and correlation based interaction networks. Major efforts on data-driven network reconstruction and model development have been centered on input/output-based and probabilistic graphical models. Input/output-based approaches are less tedious compared to probabilistic graphical models. Some of the contributors to input/output-based modeling include Bonneau et al. [10], Janes et al. [11] and Pradervand et al. [12]. Among the probabilistic graphical models, Bayesian networks are most popular [13],[14]. Contributions in Bayesian network-based modeling include the work of Sachs et al. [15], Hartemink et al. [16] and Yu et al. [17]. As discussed in a recent review by Camacho et al. [18], many other approaches such as partial correlation analysis and other statistical and systems engineering methods have been developed [19]–[25]. These recent efforts emphasis the importance of applying systems approaches to decipher and reconstruct cellular networks using high-throughput data.

The time lag between the interactions because of localization, membrane barrier and transportation has motivated researchers to employ dynamic modeling techniques. Dynamic models and temporally causal networks are derived by mapping the input data at a previous (current) time-point to the output data at the current (future) time-point to capture the temporal causal effects explicitly [26]–[28]. Recently, many approaches have been developed for reconstructing networks using dynamic (time series) data. These include (1) state-space representation based techniques [10],[29],[30] and (2) dynamic Bayesian networks [31],[32]. Such networks have been more efficient in predicting significant connections reported in the available literature. Most approaches intended for utilizing dynamic data for network identification can also handle steady-state data by setting the time-rate of change of the output or the state nodes to zero [10].

In the present work, we have applied the linear regression approach (input/output modeling) and statistical hypothesis testing to infer the important connections amongst signaling pathways using phosphoprotein time-series data. Partial least squares (PLS) method is used for input/output mapping (linear regression). The advantage of using PLS is that it calculates principal components (PCs) in the direction of output and captures sufficient variation in the output data with relatively lesser number of PCs or latent variables [33]–[35]. F-test and t-test are used for statistical hypothesis testing. This manuscript is organized as follows. In the next section, we present the results and validation of the model followed by discussion. The last section briefly discusses the experimental data preprocessing and the methodology used for the network reconstruction.

## Results

### Correlation Map

**Figure 1:**
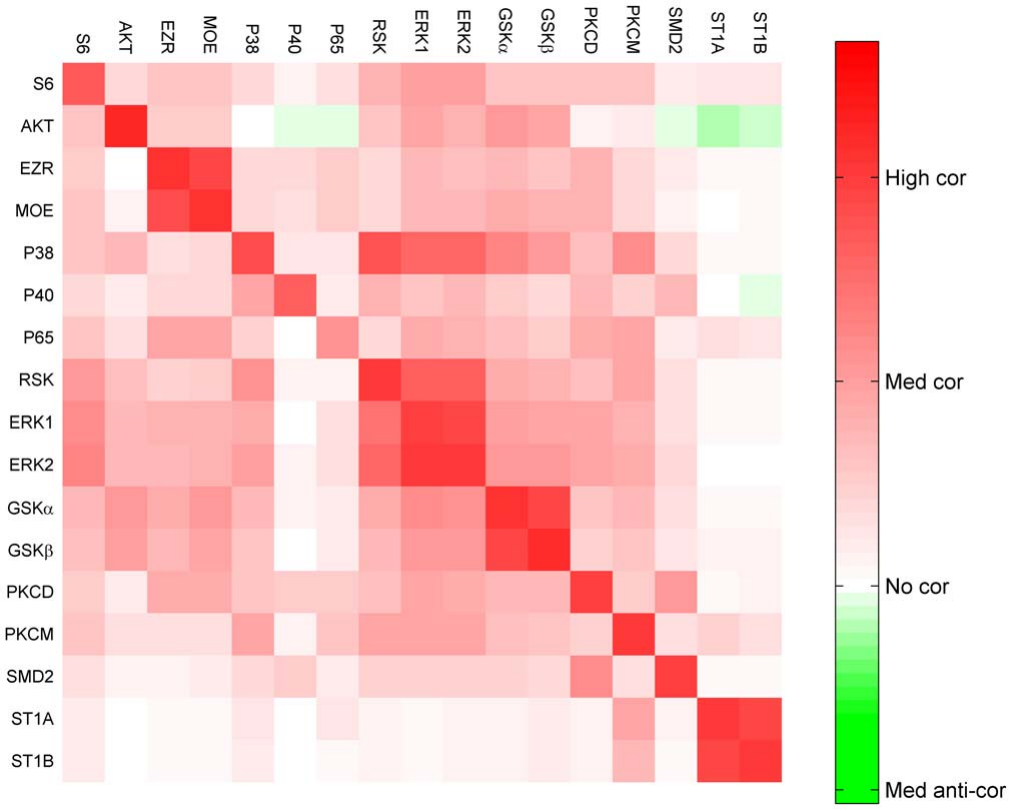
Heat-map of the correlation matrix between the input and the output variables. The rows and columns correspond to inputs and outputs, respectively. Negative values of the correlation are small in magnitude (e.g., AKT to P40) compared to positive values of the correlation. Hence, to enhance the visualization, asymmetric color-scale is used.

The potential relationships were inferred using the correlation between the input (predictor) variables and the output (response) variables. The correlation matrix between the input and output data is visualized using heat-map in Figure 1. The rows and the columns represent the inputs (PPs at t_k−1_) and outputs (PPs at t_k_), respectively. Please see Table 1 for the names of the PPs. High correlation was observed along the diagonal in Figure 1 with the indication that most of the phosphoproteins were highly self-regulated. The high self-correlation of PPs can be explained from the fact that most of the chosen PPs are from independent signaling pathways in this study and majorly activated by their upstream signaling molecule rather than by interaction/cross-talk between pathways. Thus in our modeling approach, we have also allowed this possibility via self activation of its phosphorylation. PPs in the same pathways showed high correlation. For example, ERK1/2 and RSK are the part of classical map kinase pathway and showed high correlation with each other. Variants of the same PP (i.e. GSKα/β and ST1A/B) and the member of the same family (EZR and MOE: part of ERM family) also show high correlation. The PPs belonging to independent pathway (e.g. P40 and ST1A/B) showed no correlation with most of other PPs. In this dynamic correlation matrix, high correlation was also observed from P38 to ERK1/2 and its downstream target RSK. We did not observe good correlation from ERK1/2 to P38, which suggested that there is a directed edge from P38 to ERK1/2 but not vice versa. The isoforms of protein kinase C (PKCD and PKCM) also did not show good correlation with each other indicating that they are regulated differently.

**Table 1:** The names of the 22 ligands used and the phosphoproteins measured in the AfCS experiments.

Ligands	Abbreviations
2-Methyl-thio-ATP	2MA
Resiquimod (R-848)	848
Complement C5a	C5A
Granulocyte macrophage colony stimulating factor	GMF
Interleukin-4	I04
Interleukin-6	I06
Interleukin-10	I10
Interleukin-1-beta	I1B
Interferon-alpha	IFA
Interferon-beta	IFB
Interferon-gamma	IFG
Isoproterenol	ISO
Lysophosphatidic acid	LPA
Lipopolysaccharide	LPS
Macrophage colony stimulating factor	MCF
PAM2CSK4	P2C
PAM3CSK4	P3C
Platelet Activating Factor	PAF
Prostaglandin E2	PGE
Sphingosine-1-phosphate	S1P
Transforming growth factor-beta	TGF
Uridine 5′-diphosphate	UDP

Phosphoproteins	Abbreviations
Protein kinase B (Akt)	AKT
Ezrin/Radixin	EZR
Moesin	MOE
Protein kinase C	PKC
Nuclear factor kappa beta (NFkB) p65	P65
Mitogen activated protein (MAP) kinase	MAPK
p38 MAP kinase	P38
p90 ribosomal S6 kinase	RSK
Extracellular signal-regulated kinase (Erk)	ERK
Glycogen synthase kinase 3	GSK
Signal transducer and activator of transcription 1 (STAT1)	ST1
Ribosomal protein S6	S6
Mothers against decapentaplegic homolog 2 (SMAD2)	SMD2
Neutrophil cytosolic factor 4 (p40)	P40

### Identification of Phosphoprotein Signaling Network

**Figure 2:**
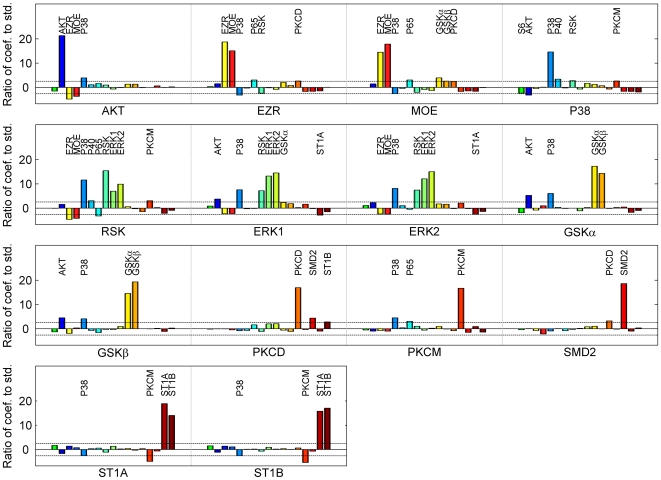
Identification of statistically significant phosphoproteins (acting as inputs) in the regulation of the signaling pathways (PPs acting as outputs). The labels on the X-axis are the names of the output PPs. For each output PP, one bar is drawn for each input PP. The Y-axis represents the ratio of the coefficient for the input in the actual model to the standard deviation of the corresponding coefficients in the random models. Thus, Y-axis is equivalent to z-score. The horizontal dashed-line denotes the threshold, 2.58 on the ratio, corresponding to 99% confidence level. The bars crossing the threshold line represent the statistically significant interaction. The names of the inputs with the absolute ratio-value greater than 90% of the threshold are also listed for each input.

The PLS models were developed using Eq. 2. Quality control of the model was performed at two steps: (1) during the PLS model development (at least 50% variance in output data captured) and (2) during the K-fold cross-validation. Overall, only 14 phosphoprotein models were selected out of 17 phosphoproteins (S6, P40 and P65 were rejected). Table S1 in Supporting Information lists the number of PCs used and the percentage variance captured in each PLS model. Significant interactions were deciphered using the statistical method discussed in the Materials and Methods section. The method of PLS is preferred for input/output modeling because of its ability to handle noise and dependency in the data and to reduce the dimension of the linear equations. Figure 2 shows the plot of the ratio (*r*) of the coefficient-values in actual model to the standard deviation of the corresponding coefficient-values in random models for each input for selected outputs. We have omitted those output variables whose model was not valid under the selection criteria described in the model development and cross-validation. The significant interactions are identified by applying the threshold on *r*. Only a few of interactions are discovered as being significant per model and capture the significant variation in the output variable. Names of PPs with significant coefficients and possible significant coefficients (90% of the threshold) are shown in Figure 2.

### Minimal Models and Their Predictive Power

**Figure 3:**
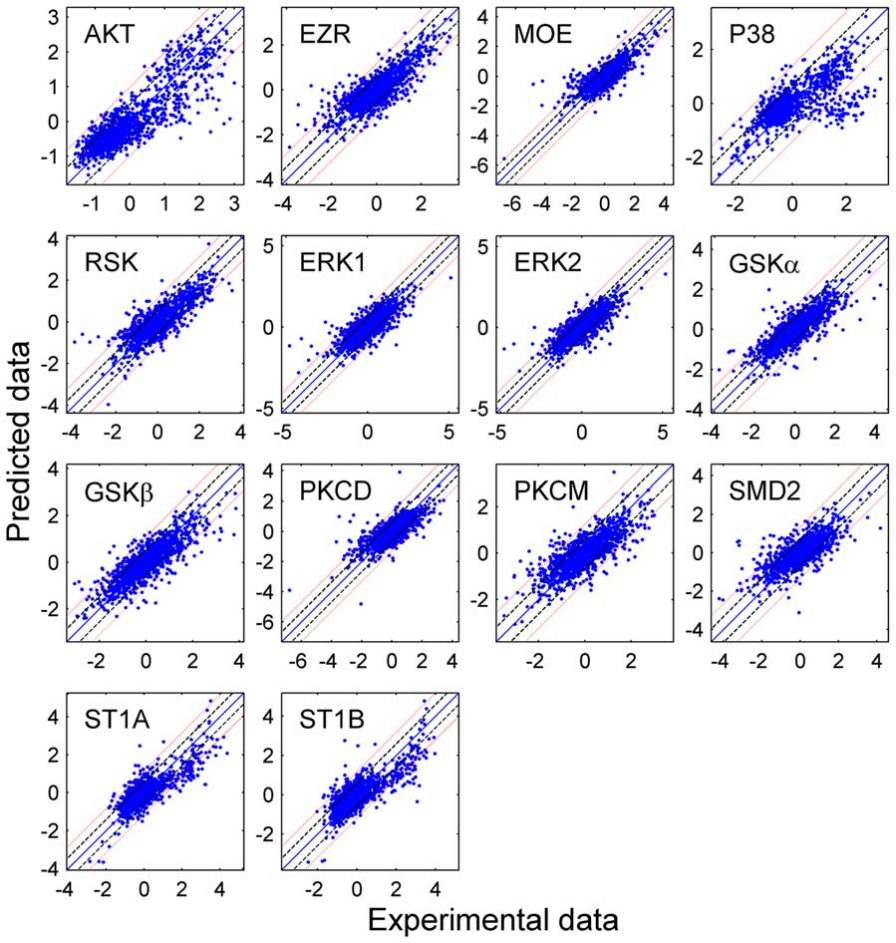
Predictive power of the reduced models containing only significant predictors. The X- and Y-coordinates represent the experimental and predicted values, respectively. The central diagonal line is the y = x line (i.e. perfect fit with no residual error). The dashed and dotted lines, denote the y = x±σ_f_ and y = x±2σ_f_ lines, respectively; where σ_f_ is the fit-error between the experimental data and the prediction made by the full model that included all the inputs and was obtained by linear-regression.

The minimal model was constructed using only the significant connections identified from Figure 2. For comparison with the PLS models, the percentage variance captured by the minimal models is listed in Table S1 in Supporting Information. To check the validity of the minimal model, we also developed the full model containing all variables as inputs. The root mean square error was calculated for the minimal model (σ_r_) and the full model (σ_f_). Figure 3 shows a scatter-plot between experimental measurements versus the predicted outputs for the minimal models at a future time-point. Solid lines (y = x), the dashed line (y = x±σ_f_) and dotted line (y = x±2σ_f_) were drawn to show the fit to the experimental data. Most of the data points lie within the ±2σ_f_ band and hence, the reduced model is a good predictive model. F-test was conducted to verify the similarity in the prediction-error between the reduced model (σ_r_) and the full model (σ_f_). It was successful with 95% confidence level for all valid models.

### Reconstructed Network and its Graphical Properties

**Figure 4:**
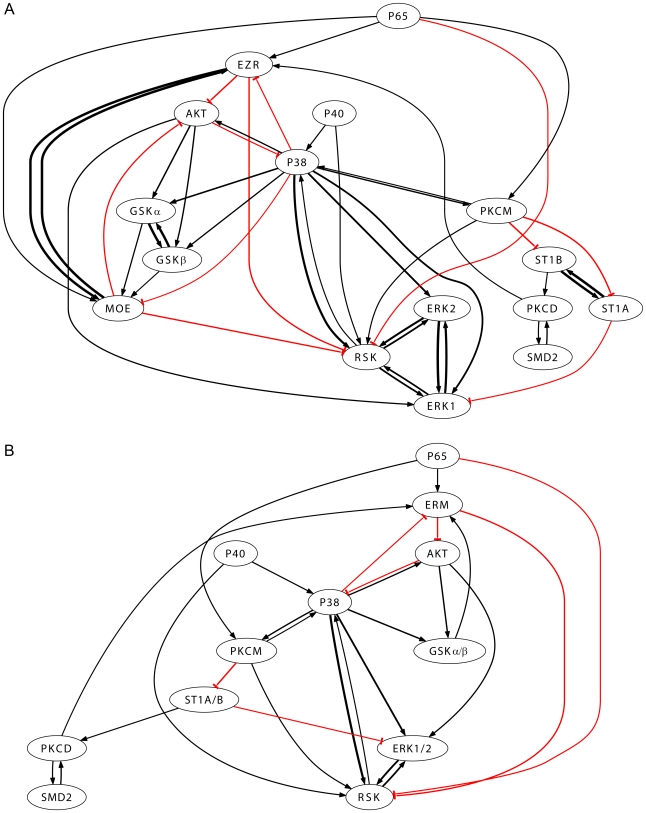
Reconstructed phosphoproteins signaling network in RAW 264.7 macrophages. (A) Full network with 16 phosphoprotein nodes. Activation and inhibition are shown with black (arrow end) and red (blunt end) lines, respectively. Thickness is proportional to (square root of) the confidence in that interaction. (B) Short network after combining ERK1 and 2, GSKα and β, ST1A and B, and EZR and MOE in single node. To do so, the corresponding rows and columns in the matrix of significant ratios were averaged without any ambiguity in the signs of the incoming or outgoing edges.

Figure 4A represents the combined network obtained from the models. The thickness of the interaction lines is proportional to (the square root of the) confidence obtained from statistical analysis. Strong similarity was observed between correlation coefficients and the significant PLS regression coefficients for most of the phosphoproteins. Examples include AKT → GSKα and β, ERK1 and 2 ↔ RSK and PKCD ↔ SMD2. Variants of the same PP (i.e. ERK1 and 2, GSKα and β, and ST1A and B) and the member of the same family (EZR and MOE: part of ERM family) showed strong significant interaction with each other and similar interaction with other PPs. Thus for the clarity of presentation, we have combined these nodes into one node, e.g. ERK1 and 2 were combined into ERK1/2, GSKα and β in GSKα/β, ST1A and B in ST1A/B and EZR and MOE in ERM (Figure 4B). It is important to note that since dynamic mapping is used to reconstruct the signaling network, the resulting network inherently captures the temporally causal connections.

The reconstructed network was characterized with respect to commonly used graph-theoretic metrics defined in the Materials and Methods section. For all pairs of distinct nodes in the network of Figure 4B, all the directed paths between them were identified using a depth-first search approach [36] (for full list of all the paths, refer Table S2 in Supporting Information). As an example, Table 2 lists all the paths from P38 to GSKα/β. From this, the total number of paths, the shortest path length and the average path length for the pairs of nodes are computed (Table 3). Given that there are some false-positive connections (correlation can result in false reversible connection) in the network, these measures are slightly biased. However, they are still useful because they provide a lower-bound (for the minimum path-length) and upper-bound (for the average path-length) and usually exclude totally unrelated interactions. In the example shown in Table 2, P38 affects GSKα/β directly and indirectly via AKT. It is difficult to predict the true paths but they do assist in formulating hypotheses for further investigation through specific knockout or inhibition experiments. P38 has the highest degree and acts as a hub. The implication is that any perturbation to P38 will affect many downstream targets/pathways.

**Table 2:** All paths from P38 to GSKα/β.

Path length	Path (→ positive edge;⊣ negative edge)
2	P38 → AKT → GSKα/β
3	P38 ⊣ ERM ⊣ AKT → GSKα/β
1	P38 → GSKα/β
6	P38 → PKCM ⊣ ST1A/B → PKCD → ERM ⊣ AKT → GSKα/β

**Table 3:** Graph-theoretic metrics for the network (Figure 4B).

	Property	AKT	ERM	P38	RSK	ERK1/2	GSKα/β	PKCD	PKCM	SMD2	ST1A/B
**AKT**	# paths		7	3	9	11	3	3	3	3	3
	MPL		2	1	2	1	1	4	2	5	3
	APL		4	2.7	3.4	4.2	2.3	5.7	3.7	6.7	4.7
**ERM**	# paths	2		3	6	9	5	3	3	3	3
	MPL	1		2	1	2	2	5	3	6	4
	APL	2		2.7	3.5	3.7	3.4	5.7	3.7	6.7	4.7
**P38**	# paths	4	4		12	12	4	1	1	1	1
	MPL	1	1		1	1	1	3	1	4	2
	APL	2.8	2.5		3.5	3.5	3	3	1	4	2
**P40**	# paths	8	8	2	13	19	8	2	2	2	2
	MPL	2	2	1	1	2	2	4	2	5	3
	APL	4.3	4	1.5	4.2	4.6	4.5	4.5	2.5	5.5	3.5
**P65**	# paths	17	15	10	21	36	23	5	5	5	5
	MPL	2	1	2	1	2	3	3	1	4	2
	APL	5	4.7	4.3	4.8	5.1	5.5	5.6	3.6	6.6	4.6
**RSK**	# paths	4	4	1		7	4	1	1	1	1
	MPL	2	2	1		1	2	4	2	5	3
	APL	3.8	3.5	1		3.7	4	4	2	5	3
**ERK1/2**	# paths	4	4	1	1		4	1	1	1	1
	MPL	3	3	2	1		3	5	3	6	4
	APL	4.8	4.5	2	1		5	5	3	6	4
**GSKα/β**	# paths	2	1	3	6	9		3	3	3	3
	MPL	2	1	3	2	3		6	4	7	5
	APL	3	1	3.7	4.5	4.7		6.7	4.7	7.7	5.7
**PKCD**	# paths	2	1	3	6	9	5		3	1	3
	MPL	2	1	3	2	3	3		4	1	5
	APL	3	1	3.7	4.5	4.7	4.4		4.7	1	5.7
**PKCM**	# paths	11	10	6	14	20	14	1		1	1
	MPL	2	2	1	1	2	2	2		3	1
	APL	4.5	4.2	4	4.2	4.5	5.1	2		3	1
**SMD2**	# paths	2	1	3	6	9	5	1	3		3
	MPL	3	2	4	3	4	4	1	5		6
	APL	4	2	4.7	5.5	5.7	5.4	1	5.7		6.7
**ST1A/B**	# paths	5	4	4	6	8	8	1	4	1	
	MPL	3	2	3	2	1	4	1	4	2	
	APL	4.6	4.3	4.3	4.5	4.8	5.3	1	5.3	2	

The rows and columns correspond to start and terminal nodes, respectively. For each row-node and column-node pair, the total number of paths, minimum path-length (MPL) and average path-length (APL) are listed. The average path-length for the whole network, defined as the average of shortest path-length between all pairs of nodes, is 1.83. Average degree of the nodes is 4.3.

## Discussion

Phosphoprotein signaling is important in modulating various functions. Thus the understanding of the interaction/cross-talk between them is crucial. Here, we reconstructed the phosphoprotein signaling network that captures the interactions known in the literature and also suggests novel interactions. These models reasonably predict the interactions between phosphoproteins. Interactions of these phosphoproteins have been studied extensively. Therefore, the reconstructed network (Figure 4) was validated from information available in the literature.

### 

#### Significant signaling pathways

AKT is widely known for GSK phosphorylation and inhibiting its activity [37]–[39]. The reconstructed network captured the same outcome i.e. phosphorylation of GSK via AKT, thus inhibiting its activity. However, the convention used in the reconstructed network differs from the literature. Here positive and negative connections represent activation and inhibition of phosphorylations whereas positive and negative connections in literature are based on activation and inhibition of the activity of molecules.

The reconstructed map captured the positive connectivity between ERK and RSK, members of the classical MAP kinase pathway [37],[40]. A positive connectivity was also found from PKCM to RSK. This relationship can be a resultant of partial artifact of the mathematical technique, PLS, used in the network reconstruction. PLS captures correlation between input and output variable. According to the literature, PKC activates ERK[41] which subsequently activates RSK through MAP kinase pathway[40]. Thus, RSK is supposed to have a good correlation with PKC and was captured in modeling. In general whenever there is connection A → B, all correlation based modeling techniques produce bidirectional connections (A ↔ B). Such false positives can only be partially removed by dynamic modeling/time mapping as used here. PKCD ↔ SMD2 was observed, whereas literature showed only PKC → SMD2 [42],[43]. Similarly, PKCM ↔ P38 was observed instead of PKCM → P38 [44]. This reconstruction also captured other known relationships including PKCD → EZR/MOE [45],[46] and P38 → GSK[47]. There were other observed correlations which exhibited reverse connections than expected. For example, existing research suggests the connections PKCM → P65 [48],[49] whereas our model captured the reverse connections P65 → PKCM. This observation was attributed to an outcome of PLS methodology as explained above.

#### Correlated groups/proteins

The variants of the same protein are mostly regulated similarly. The high correlation was observed for these variants GSK α and β, ERK1 and 2 and ST1A and B. However, this was not observed for PKCD/M insinuating different regulatory pathways for the two variants of PKC. EZR and MOE are the members of Ezrin/radixin/moesin (ERM) pathway and commonly involved in regulation of actin cytoskeleton [37]. Consequently, a high correlation was observed between them as expected.

#### Rejected models (PPs with no input in the network)

The models for P40, P65 and S6 were rejected. Hence, they do not have any inputs in the network of Figure 4B. Rejected model PPs have either no interaction with other pathways or they interact with other molecules/phosphoproteins not measured in the AfCS dataset. For example, P40 is a regulatory component of the superoxide-producing phagocyte NADPH-oxidase pathway [37],[50]. We did not find any relevant interaction for P40 in the literature. P65 is regulated by other factors such as IκB kinases (IKK) which are not measured in the AfCS dataset [51]. Similarly, S6 is regulated by both p90RSK and p70RSK [52]. However, only p90RSK is measured in the AfCS dataset. The correlation between p90RSK and S6 is positive in Figure 1. However, this does not result in any significant connection in the network.

This modeling approach also provides support for investigating novel or less studied interactions. For example, a high correlation between P38 → RSK was also observed in Figure 1 and this interaction was captured with high confidence in the reconstructed network (Figure 4). Some evidence has been found underpinning this interaction [53], but most of the reviews do not discuss about it explicitly [40],[54]. Similarly, GSK → EZR/MOE connection was observed in the reconstructed network. There is some indirect evidence about this relationship through PI3 kinase [37],[55], but a direct relationship has not been reported and merits further investigation. Lastly, a few inhibitory connections were found in this network. Several of these have not been reported in the literature. If these are false positives, there can be two possibilities, both being mathematical artifacts. First, the degradation term is not included in the linear modeling approach. Second, the inclusion of the self regulation term which receives very high weight (coefficient) is possibly altering the coefficients for the other inputs (described in the Materials and Methods section).

**Figure 5:**
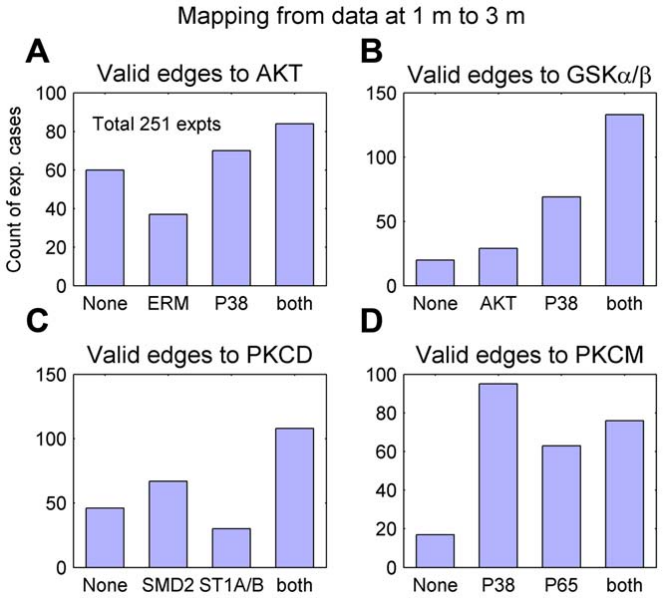
Distribution of the consistency of pathways for nodes which are activated through two different pathways. X-axis represents four possibilities: (1) neither path consistent (‘None’), (2) path 1 consistent, but not 2 (3) path 2 consistent but not 1, (4) both paths consistent (see text). Y-axis represents the number of experiments counted for each case.

The nodes with in-degree greater than 1, which have multiple pathways for activation, were further investigated for the relative importance of the incident edges/connections. Figure 5 shows the distribution of experimental cases for the activation of select nodes with in-degree of 2. Such examples include AKT, GSKα/β, PKCD and PKCM. The purpose is to observe when a selected node is activated (protein is phosphorylated), then whether this activation is through input from only one of the two upstream nodes, or signaling through both the paths is required (labeled ‘both’). There exists a fourth possibility that none of the two paths are consistent with the observed state of the target node (labeled ‘None’), suggesting that other edges, involved in unmeasured pathways or involved in measured but not captured in our network, are contributing to the activation of the node. An edge from node i to node j is consistent if sign(node i)*sign(edge(i→j)) = sign(node j). The analysis of AKT (Figure 5A) indicated that for most experimental cases (ligand combinations), AKT required input either from only P38 or from both P38 and ERM. There are significant number of cases in which the signal flows through neither path was consistent with the state of AKT (labeled ‘None’ in Figure 5A). This clearly indicated that AKT is also being activated via pathway(s) other than the measured phosphoproteins. In the case of GSKα/β, most of the cases are captured via either activation from P38 or activation from both P38 and AKT (Figure 5B). There are only few cases where activity of GSK is in concordances with regulation via AKT or none. Similar interpretation can be drawn for PKCD and PKCM from Figure 5C and D.

**Figure 6 pcbi-1000654-g006:**
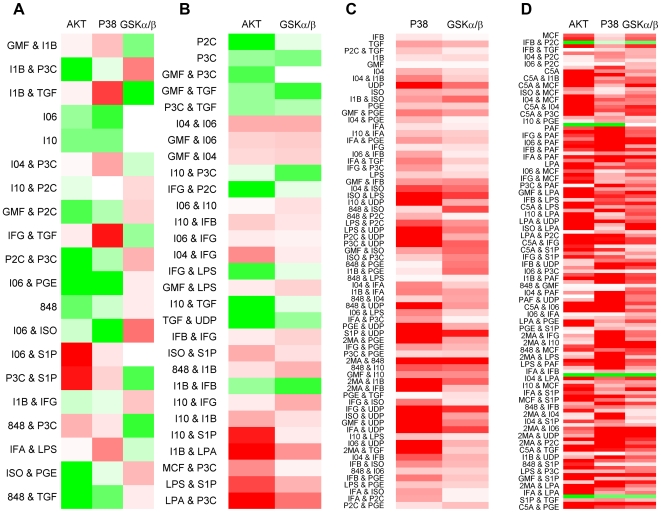
Display of experimental data corresponding to the four cases of valid paths from P38 and/or AKT to GSKα/β in Figure 5B. Red and green colors are for positive and negative values (log(*x*), where *x* is ratio of raw value at current time to the raw value at *t* = 0), respectively, with the darker color indicating larger magnitude. (A) None of the two edges are valid because the level of GSKα/β is changing in opposite direction to what the signals through the paths P38 → GSKα/β or AKT → GSKα/β would otherwise result in. (B) The effective signaling is through the path AKT → GSKα/β alone. (C) Only the path P38 → GSKα/β is valid. (D) Both paths are valid as indicated by the same color in each column for all rows. 1 min data was used for AKT and P38. 3 min data was used for GSKα/β.

**Figure 7:**
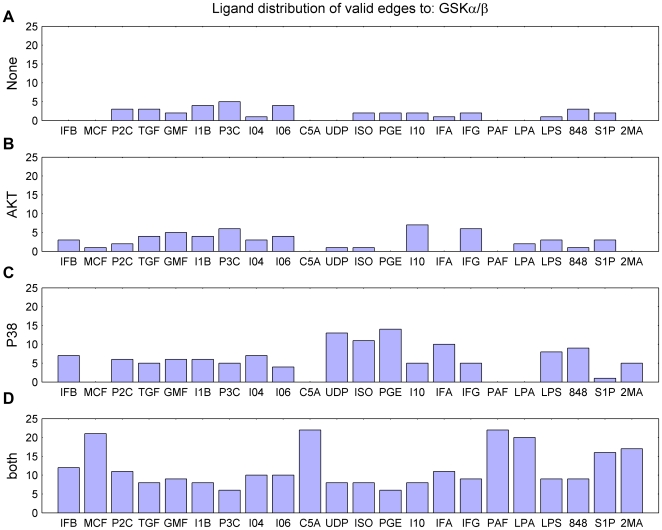
Ligand distribution for all four cases of GSKα/β activation (discussed in Figure 5B). X-axis and Y-axis represent the name of ligand and counts of the cases, respectively. For dual ligand experiments, the case is added to both of the ligands. The panels A–D also correspond to the heat-maps of Figure 6 A–D, respectively.

The activation of GSKα/β was further probed for the different ligand combinations. To do this, the experimental data corresponding to the four cases of consistency of the edges from AKT and P38 to GSKα/β (Figure 5B) is displayed as a heat-map in Figure 6. In the first case (A), none of the edges are valid because the level of GSKα/β is changing in opposite direction to what the signals through the paths P38 → GSKα/β (activation) or AKT → GSKα/β (activation) would otherwise result in. As an example in the experiment of double ligands GMF (Granulocyte macrophage colony stimulating factor or GMCSF) and I1B (IL-1B), both AKT and P38 are positive at *t* = 1m whereas GSKα/β is negative at *t* = 3m. In the second case (B), the validity of the signal from AKT → GSKα/β is evident from the data analysis, as seen in Figure 6B. In the third case (C), interestingly, P38 and GSKα/β are both positive in all such experiments and naturally AKT is negative. Finally, in the fourth case (D), strong phosphorylation of GSKα/β is observed since the signals through both the paths superimpose constructively in most of the experiments. Similar heat-maps for the response nodes (phosphoproteins) AKT (Figure 5A), PKCD (Figure 5C) and PKCM (Figure 5D) are displayed in Figures S1, S2, and S3, respectively (Supporting Information). Then, we plotted a bar-graph of ligand distribution for all the four cases (Figure 7). With such an elaborate yet intricate display, one can easily see that the ligands MCF, C5A, PAF and LPA signal through both P38 and AKT to phosphorylate GSKα/β [47],[56]. In fact, the data indicates that MCF and LPA signals through AKT alone in only one case (namely, with P3C) and two cases (namely with P3C and I1B), respectively, out of 22 possible combinations with self or other ligands. The ligands S1P and 2MA also transduce the signal through both pathways in most cases. On the contrary, the ligands UDP, ISO, PGE and IFA lead to phosphorylation of GSKα/β only through the P38 pathway (Figure 7C). This observation leads to the hypothesis that inhibition of P38 would reduce or abolish phosphorylation of GSKα/β. This can be tested by using specific inhibitors of P38 (SB203580 [57] or SB202190 [58]) for a specific ligand such as PGE (see Figure 7C). It is interesting to note that one can arrive at conclusions about specific ligands, phosphoproteins or pathways from global or systemic analysis.

The predictor-response (or input-output) characteristics for the other three phosphoproteins, namely, AKT, PKCD and PKCM, analyzed in Figure 5 can be further studied with respect to the various experiments using the ligand distribution for different valid paths displayed in the bar-graphs of Figures S4–S6, respectively (Supporting Information).

#### Explanation of complex scenario with multiple connections

Several output nodes in the network have multiple input connections. Depending upon the state of the predecessor nodes, these connections can affect the output node in the same or opposite directions. The reconstructed network can be used to understand/explain such cases. For example, in Figure 4B, GSKα/β is phosphorylated/affected by both P38 and AKT (P38 → GSKα/β, AKT → GSKα/β). In turn, AKT, is affected by P38 (P38 → AKT) and ERM (ERM ⊣ AKT) (see also Figure 5A, Figure S1 and Figure S4). Now, in the context of interpreting/projecting data for P38, AKT and GSKα/β, it is possible that for some ligand inputs only the edge AKT → GSKα/β is valid (Figure 5B and Figure 6B). This can happen if the effect of the edge ERM ⊣ AKT dominates over that of the edge P38 → AKT. Another case, where only the edge P38 → GSKα/β is valid (Figure 6C), is more intriguing. This complexity arises because the positive edge P38 → AKT, P38 tries to set the state of AKT in its direction. Intuitively, this makes one think that AKT should have the same sign as P38 and the edge AKT → GSKα/β should be valid. However, in our experimental data there exist cases with only P38 → GSKα/β as a valid edge. This anomaly can be explained by including the node ERM in the analysis.

The sign of AKT is set by both the sign of P38 and ERM. Hence, the sign of ERM can be considered to explain the cases of only the edge P38 → GSKα/β being valid. One such case is the application of the ligands IL-4 (I04) and IL-1b (I1B) (Figure 6C, 7th row from top, both P38 and GSKα/β are positive). In Figure S1B (valid edge ERM ⊣ AKT, 2nd row from top), ERM is positive and AKT is highly negative. This case actually suggests that interpretation of the individual nodes locally has limited scope and complete interpretation is possible only at a systemic level.

As seen above, interpretation of individual experimental sets is complicated. Even more complications arise if one or more of the inputs are unmeasured. One can imagine a scenario where protein A is affected by protein B which is also affected by unmeasured protein C. Two interesting scenarios can arise depending upon the effect of protein C on protein B: (1) B increases without increase in A, if the effect of C on B is positive and (2) A increases but B does not, if the effect of C on B is negative. Such cases can be explained similar to the above examples if we hypothesize some input nodes to be unmeasured.

#### Prediction capability for extrapolation

**Figure 8:**
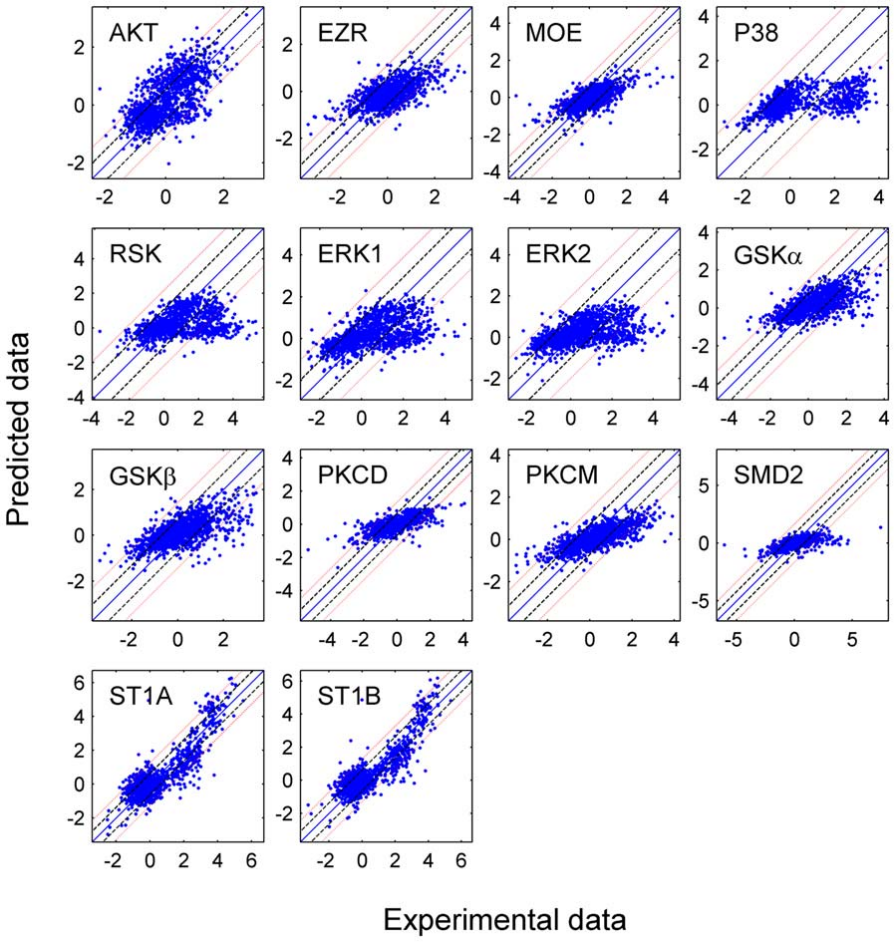
Prediction of data at 10 min using the experimental data at 3 min and the reduced models containing only significant predictors. Numerical integration is used to eliminate the error due to discrete approximation of time-derivatives. The central diagonal line is the y = x line (i.e. perfect fit with no residual error). The dashed and dotted lines, denote the y = x±σ_f_ and y = x±2σ_f_ lines, respectively; where σ_f_ is the best (minimum) fit-error obtained by linear-regression between the experimental data at 3 min (as input) and the experimental data at 10 min (as output).

To test the ability of the network of Figure 4 to predict data at 10 min using data at 3 min, we have performed the following study:

1. We have used the models developed from 1 min and 3 min data to predict the data at 10 min using the experimental data at 3 min. The results are shown in Figure 8 and Table S3 in Supporting Information. These results show that the models developed using the 1 and 3 min data have reasonable predictive power for all of the outputs/phosphoproteins at 10 min. For extrapolation to 10 min, we have used numerical integration to avoid the error due to discrete approximation of time-derivatives. The phosphoprotein levels at 10 min were calculated using the pseudo-rate parameters obtained from the model (from 1 min and 3 min data) and initial state at 3 min. Table S3 also lists the fraction of data points which lie within the σ and 2σ band in Figure 8. Similar numbers for the prediction of 3 min data in Figure 3 are also listed in Table S3 for comparison. The predictive power for extrapolation is better for some of the PPs such as SMD2 and ST1A/B as compared to others such as AKT and P38. This is likely because SMD2 and ST1A/B translocate into the nucleus and are dephosphorylated only in the nucleus [59],[60]. Hence, their phosphorylation can sustain for longer time and result in better agreement between the experimental data and the model predictions at 10 min.
2. We performed pathways-based advanced statistical analysis similar to what is shown in Figures 5–7 and S1, S2, S3, S4, S5, and S6, using the mapping of data from 3 min to 10 min. The results are shown in the Figures S7, S8, S9, S10, S11, S12, S13, S14, and S15 (Supporting Information). It is evident that despite the differences between the individual cases, the overall broad statistical features are similar between the mappings from 1 min to 3 min and from 3 min to 10 min. This suggests that most of the connections in Figure 4 are qualitatively correct.

In conclusion, deciphering the interconnectivity of large signaling networks is a complex problem in systems biology. The analysis and interpretation of large-scale temporal datasets on such pathways is challenging due to myriad issues such as multiple input-multiple output connectivity and lack of temporal data at appropriate time-scales. In the present work, a novel algorithm for the discovery of bio-molecular networks using large-scale temporal data is presented. Our methodology integrates partial least squares based dynamic input/output (state-space) modeling and statistical significance testing. The resulting models have sound predictive power validated by F-test. When applied to the early-time phosphoprotein dataset on RAW 264.7 macrophage cells provided by the AfCS, the approach is able to find most of the known connections between the signaling pathways. Example includes AKT → GSK. The model developed here is able to predict the 10 min data set with good accuracy. Potentially novel connections also have been found; an example is P38 → RSK. By varying the threshold of the statistical significance test, a researcher can probe additional connections which could have been otherwise missed. Thus, both the algorithmic approach and results are capable of generating novel hypothesis for further investigation. The proposed approach tackles the challenge of capturing dynamics and temporal causality in intracellular signaling pathways. The use of PLS in our approach makes it extensible to even larger data sets comprising of hundreds or thousands of measured variables. Further, these quantitative models can be used to make predictions about the response at a future time-point using the values at the current and past times.

## Materials and Methods

### Experimental Data

The experimental data was generated by the Alliance for Cellular Signaling (AfCS) laboratories using the RAW 264.7 cells. Single- and double-ligand screening for 22 ligands (Table 1) in 251 experiments were performed. In each experiment, time-dependent changes in the phosphorylation-level of 21 signaling proteins were measured at 1, 3, 10, 30 minutes after stimulation using phosphoprotein-specific antibodies (western blot analysis, AfCS protocols #PP00000177 and #PP00000181 [61]. Most experiments were done in duplicates and triplicates. For complete details of the data and the experimental protocols, please refer the AfCS website [62]. The PP data is available from the AfCS Data Center (“RAW 264.7 ligand screen” and “RAW 264.7 two-ligand screen”). The 22 ligands used and the phosphoproteins measured in experiments are listed in Table 1.

### Data Processing

Time scale for most of the signaling events lies in minutes. Response at early times, such as five minutes after the stimulation with the ligand(s), shows the primary effect of ligands stimulation and signaling from upstream (phospho)protein to the expected pathways. In most cases, the strength of an early response is much higher than the degradation rate of the signaling molecule. Contrary to this, during the later phase, the desensitization/internalization of the cell surface receptors decreases/stops the effect of ligand stimulation. The changes in the signaling molecular concentrations observed at those times are primarily due to its self degradation. Here, in the case of phosphoprotein data, degradation mostly represents dephosphorylation of the phosphoprotein. Thus, to avoid such false positive results in linear modeling approach, we have used the early time point data at only 1 and 3 minutes and ignored the later time point data at 10 and 30 minutes. However, the data at 10 min has been used for accessing the predictive power of the model. The data at t = 0 minute was not included as it was always equal to 1 (available as fold-change from the AfCS data center). For the purposes of mathematical treatment, replicates are considered as separate conditions. Explicit use of replicates has two advantages: (1) different experiments were repeated different number of times, so separate treatment of replicates resulted in more weighting to the experiments with more replicates and (2) the effect of experimental variation was automatically accounted in the statistical analysis during model development and cross validation. Data was log2 transformed prior to the modeling to give equal weighting to up- and down-regulation. Both input and output data were normalized with respect to the mean and standard deviation of the respective variables.

### Network Identification

#### Model development

A linear model was developed using partial least square (PLS) method. Matlab was used for the calculation [63] and Graphviz software was used to draw the network [64]. Dynamic mapping (y_t_ = f(y_t−1_)) was used to calculate the interaction coefficients. The model (y_t_ = f(y_t−1_)) represents the dependence of the level of measured PPs at time t on the level of all measured PPs at time t−1. If the mapping function (f) is linear, then it can be derived from the state-space representation of the system as follows.
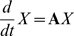
(1)After discretization and rearrangement, we get 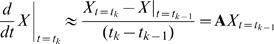, or equivalently,
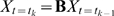
(2)Where X (the vector of state variables) denotes the set of PPs. **A** and **B** are coefficient matrices with suitable relationships between the elements of **A** and **B**. From here on, we will work with Eq. 2.

In a signaling pathway, signaling occurs from upstream molecules to downstream molecules. The limitation of this data set is that, in each signaling pathway, usually only one PP is measured. To circumvent this limitation, we have assumed that the concentration of upstream signaling molecule is same or linear function of the measured PPs (validity of this assumption is also evident from the correlation plot of Figure 1). This assumption also presents complication because (1) the rate of degradation of PP is usually proportional to its level of phosphorylation, and (2) the input effect from the upstream (phospho)protein in the same pathway is also proportional to the level/amount of the upstream protein, thus making their separation difficult.

Even though PLS has the capability to handle multiple outputs, we developed the models for individual PPs (single output). This is motivated by a criteria for model selection, namely, the input matrix should capture significant variance in the output. Only those models were selected for network reconstruction for which the input data captured at least 50% variance of the output data. The PPs for which the variance captured was less than 50% were not included in the network reconstruction with the reasoning that they are mainly regulated directly by the ligands or other pathways and molecules rather than through the measured PPs (signaling pathways) in the experiments. The number of PCs for model development was selected based on the criteria that variance captured by that last PC should capture at least 5% of total variance captured by all PCs.

#### Cross-validation

The K-fold cross-validation method was used to evaluate the performance of the proposed phosphoprotein network; for each output we find out if the fit-error of the model for the K-fold test data sets is statistically similar to that for the training set and is lesser than the fit-error for a random model for that output. The experiment data set was randomly divided into k groups. The model estimation was conducted on (k−1) groups as training set and remaining one set was used as a test set. This process was repeated until all k groups were used as a test set once. In this work, we have used k = 10, as it produces less bias, variance and sum of squared error (SSE) and has good computational efficiency [65]. For each output, mean of SSE for test set was compared with mean of SSE for training set and random models through F-test. Even though data are log2 transformed and fit-error (the difference between the predicted and experimental values) may have log-normal distribution, the use of F-test is robust as shown in ref. [66]. Detailed procedure of F-test in K-fold cross-validation is described in the Text S1 (Supporting Information). The random models were generated from the entire dataset by randomly shuffling the output as explained in the section “Statistical significance of the interaction coefficients” below. The models, for which the probability in F-test was less than 0.95 between the test and training sets and the p-value was less than 0.05 between the test set and random model, were selected for network reconstruction.

#### Statistical significance of the interaction coefficients

Significant coefficients are selected using statistical significance-test based approach. To implement the test, the probability distribution of the coefficient value for a random model is generated and compared with the coefficient value in the actual model. We use the standard deviation (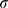) parameter of the distribution (mean value is zero). The procedure is as follows.

1. To compute 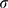, many random output datasets are generated by randomly shuffling the actual experimental output data, and PLS model is developed corresponding to each random output dataset. For a chosen output and input, 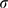 is the standard deviation of the corresponding coefficient values in the random models. If *b_r_* is the coefficient in a random model then, 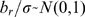, where 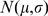 is the normal distribution with mean 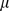 and standard deviation 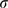.
2. Next, if *b* denotes the coefficient value in the actual PLS model for a chosen input and output, then, we compare the ratio, *r = b*/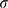, with a threshold (r_th_). r_th_ is taken as the inverse of the cumulative normal distribution function 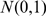 for a chosen level of confidence. A threshold (r_th_) of 2.58 corresponding to 99% confidence in two tailed t-test has been used in this study. If r>r_th_, then the connection from the chosen input to the chosen output is considered significant.

#### Graphical properties of networks

A graph, G, can be defined as a collection of nodes or vertices, 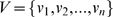, and the edges between the nodes, 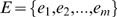. *n* is the number of nodes or the size of the graph. Thus, G = (V, E). If a direction is associated with the edges then they are called directed edges and G is called a directed graph (digraph). If the directed edge 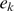 starts at the node 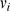 and ends at the node 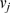 then 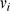 is called the start node and 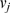 is called the terminal node of the edge 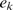, and 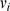 is also a predecessor node of 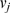. A directed path from node 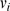 to node 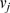 in a digraph is an alternating sequence of nodes and directed edges of the digraph (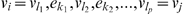) such that the edge 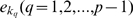 connects the node 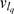 to the node 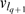, where *p* is the total number of nodes on the path [67],[68]. The number of edges on the path, i.e., path-length, is (*p*−1). The number of edges coming into (going out of) a node is called the in-degree (out-degree) of the node. The adjacency matrix of G, 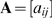, is computed as, 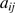 = 1, if there exists an edge from node 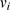 to node 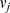, 0 otherwise. In this work, once the significant predictors are identified and the coefficient matrix **B** is computed, **A** is obtained as **A** = sign(**B** ^T^) if the sign of the edge is retained. Once **A** is known, G is completely defined.

## Supporting Information

Table S1 Number of principal components (PCs) used and the corresponding variance captured for all the phosphoproteins (outputs) in the PLS model, and the variance captured in the minimal model (using least-squares). The phosphoproteins excluded from the network were S6, P40 and P65. In most cases, the variance captured by the minimal model is slightly larger than that by the PLS model. This is because the least-squares approach is equivalent to using all the PCs in the application of PLS. To develop the PLS model, only the first few (2 or 3) PCs were used. We have verified that the variance captured by the minimal model is always lesser than by the full model if all the PCs were used.
(0.05 MB DOC)

Table S2 The list of all paths between all pairs of phosphoprotein nodes in the network of Figure 4B. The format is explained in Table 2 for all the paths from P38 to GSKA/B listed here.
(0.03 MB TXT)

Table S3 Comparison of prediction of 3 min and 10 min data using the model developed by mapping 1 min data to 3 min data. Numerical integration is used to predict the data at 10 min. σ is root mean squared error (RMSE) between the experimental data and its best linear-fit obtained between the corresponding input and output data for the two intervals independently.
(0.05 MB DOC)

Figure S1 Display of experimental data corresponding to the four cases of valid paths from ERM and/or P38 to AKT in Figure 5A.
(1.59 MB TIF)

Figure S2 Display of experimental data corresponding to the four cases of valid paths from SMD2 (SMAD 2) and/or ST1A/B (STAT 1A/B) to PKCD in Figure 5C.
(1.56 MB TIF)

Figure S3 Display of experimental data corresponding to the four cases of valid paths from P38 and/or P65 to PKCM in Figure 5D.
(1.51 MB TIF)

Figure S4 Ligands distribution for all four cases of AKT activation (displayed in Figure 5A). X-axis and Y-axis represent the name of ligand and counts of the cases respectively. For dual ligand experiment, the case is added to both of the ligands.
(0.31 MB TIF)

Figure S5 Ligands distribution for all four cases of PKCD activation (displayed in Figure 5C). X-axis and Y-axis represent the name of ligand and counts of the cases respectively. For dual ligand experiment, the case is added to both of the ligands.
(0.31 MB TIF)

Figure S6 Ligands distribution for all four cases of PKCM activation (displayed in Figure 5D). X-axis and Y-axis represent the name of ligand and counts of the cases respectively. For dual ligand experiment, the case is added to both of the ligands.
(0.31 MB TIF)

Figure S7 Distribution of the consistency of pathways for nodes which are activated through two different pathways (similar to Figure 5), based on the mapping of data from 3 min to 10 min. X-axis represents four possibilities: (1) neither path consistent (‘None’), (2) path 1 consistent, but not 2 (3) path 2 consistent but not 1, (4) both paths consistent. Y-axis represents the number of experiments counted for each case.
(0.32 MB TIF)

Figure S8 Display of experimental data corresponding to the four cases of valid paths from ERM and/or P38 to AKT in Figure S7A based on the mapping of data from 3 min to 10 min.
(1.51 MB TIF)

Figure S9 Display of experimental data corresponding to the four cases of valid paths from P38 and/or AKT to GSKα/β in Figure S7B based on the mapping of data from 3 min to 10 min.
(1.26 MB TIF)

Figure S10 Display of experimental data corresponding to the four cases of valid paths from SMD2 (SMAD 2) and/or ST1A/B (STAT 1A/B) to PKCD in Figure S7C based on the mapping of data from 3 min to 10 min.
(1.59 MB TIF)

Figure S11 Display of experimental data corresponding to the four cases of valid paths from P38 and/or P65 to PKCM in Figure S7D based on the mapping of data from 3 min to 10 min.
(1.46 MB TIF)

Figure S12 Based on the mapping of data from 3 min to 10 min: Ligands distribution for all four cases of AKT activation (summarized in Figure S7A). X-axis and Y-axis represent the name of ligand and counts of the cases, respectively. For dual ligand experiments, the case is added to both of the ligands. The panels A–D also correspond to the heat-maps of Figure S8 A–D, respectively.
(0.31 MB TIF)

Figure S13 Based on the mapping of data from 3 min to 10 min: Ligand distribution for all four cases of GSKα/β activation (summarized in Figure S7B). X-axis and Y-axis represent the name of ligand and counts of the cases, respectively. For dual ligand experiments, the case is added to both of the ligands. The panels A–D also correspond to the heat-maps of Figure S9 A–D, respectively.
(0.31 MB TIF)

Figure S14 Based on the mapping of data from 3 min to 10 min: Ligand distribution for all four cases of PKCD activation (summarized in Figure S7C). X-axis and Y-axis represent the name of ligand and counts of the cases, respectively. For dual ligand experiments, the case is added to both of the ligands. The panels A–D also correspond to the heat-maps of Figure S10 A–D, respectively.
(0.31 MB TIF)

Figure S15 Based on the mapping of data from 3 min to 10 min: Ligand distribution for all four cases of PKCM activation (summarized in Figure S7D). X-axis and Y-axis represent the name of ligand and counts of the cases, respectively. For dual ligand experiments, the case is added to both of the ligands. The panels A–D also correspond to the heat-maps of Figure S11 A–D, respectively.
(0.31 MB TIF)

Text S1 F-test in K-fold Cross-Validation
(0.08 MB PDF)
