# Supplementary material for: Identification of Crosstalk between Phosphoprotein Signaling Pathways in RAW 264.7 Macrophage Cells
Source: PLoS Comput Biol. 2010 Jan 29;6(1):e1000654. doi: 10.1371/journal.pcbi.1000654 (PMC2813256; doi:10.1371/journal.pcbi.1000654)
Supplement: Table S1 — Number of principal components (PCs) used and the corresponding variance captured for all the phosphoproteins (outputs) in the PLS model, and the variance captured in the minimal model (using least-squares). The phosphoproteins excluded from the network were S6, P40 and P65. In most cases, the variance captured by the minimal model is slightly larger than that by the PLS model. This is because the least-squares approach is equivalent to using all the PCs in the application of PLS. To develop the PLS model, only the first few (2 or 3) PCs were used. We have verified that the variance captured by the minimal model is always lesser than by the full model if all the PCs were used. (0.05 MB DOC) [file pcbi.1000654.s001.doc]

| Name | PLS model (PLS on all inputs) | |  | Minimal model |
| --- | --- | --- | --- | --- |
|  | No. of PC used | % variance captured |  | % variance captured |
| S6 | 3 | 44 |  | - |
| AKT | 3 | 74 |  | 75 |
| EZR | 2 | 60 |  | 64 |
| MOE | 2 | 61 |  | 64 |
| P38* | 3 | 49 |  | 50 |
| P40 | 3 | 43 |  | - |
| P65 | 3 | 19 |  | - |
| RSK | 2 | 63 |  | 67 |
| ERK1 | 2 | 63 |  | 64 |
| ERK2 | 2 | 61 |  | 63 |
| GSKα | 2 | 65 |  | 66 |
| GSK β | 2 | 64 |  | 68 |
| PKCD | 3 | 55 |  | 56 |
| PKCM | 3 | 60 |  | 61 |
| SMD2 | 3 | 53 |  | 54 |
| ST1A | 3 | 64 |  | 65 |
| ST1B | 3 | 64 |  | 63 |

^*^: Even though the variance captured with 3 PCs is slightly below 50%, we have used this model for generating the network.
