# Supplementary material for: Identification of Crosstalk between Phosphoprotein Signaling Pathways in RAW 264.7 Macrophage Cells
Source: PLoS Comput Biol. 2010 Jan 29;6(1):e1000654. doi: 10.1371/journal.pcbi.1000654 (PMC2813256; doi:10.1371/journal.pcbi.1000654)
Supplement: Table S3 — Comparison of prediction of 3 min and 10 min data using the model developed by mapping 1 min data to 3 min data. Numerical integration is used to predict the data at 10 min. σ is root mean squared error (RMSE) between the experimental data and its best linear-fit obtained between the corresponding input and output data for the two intervals independently. (0.05 MB DOC) [file pcbi.1000654.s003.doc]

| Names | Fraction of data points within σ_i_ or 2σ_i_ band | | | | |
| --- | --- | --- | --- | --- | --- |
|  | predicted vs. exp. at 3 min | |  | predicted vs. exp. at 10 min | |
|  | σ­_1_ | 2σ­_1_ |  | σ_2_ | 2σ_2_ |
| AKT | 0.71 | 0.95 |  | 0.57 | 0.87 |
| EZR | 0.71 | 0.94 |  | 0.62 | 0.91 |
| MOE | 0.76 | 0.95 |  | 0.67 | 0.93 |
| P38 | 0.81 | 0.94 |  | 0.65 | 0.84 |
| RSK | 0.71 | 0.94 |  | 0.68 | 0.85 |
| ERK1 | 0.72 | 0.95 |  | 0.66 | 0.88 |
| ERK2 | 0.71 | 0.95 |  | 0.64 | 0.89 |
| GSKα | 0.73 | 0.95 |  | 0.62 | 0.91 |
| GSKβ | 0.73 | 0.95 |  | 0.66 | 0.92 |
| PKCD | 0.75 | 0.95 |  | 0.67 | 0.92 |
| PKCM | 0.72 | 0.95 |  | 0.64 | 0.90 |
| SMD2 | 0.75 | 0.95 |  | 0.74 | 0.93 |
| ST1A | 0.72 | 0.94 |  | 0.63 | 0.93 |
| ST1B | 0.74 | 0.95 |  | 0.66 | 0.93 |
