# Supplementary figures and images for: Identification of Crosstalk between Phosphoprotein Signaling Pathways in RAW 264.7 Macrophage Cells
Source: PLoS Comput Biol. 2010 Jan 29;6(1):e1000654. doi: 10.1371/journal.pcbi.1000654 (PMC2813256; doi:10.1371/journal.pcbi.1000654)

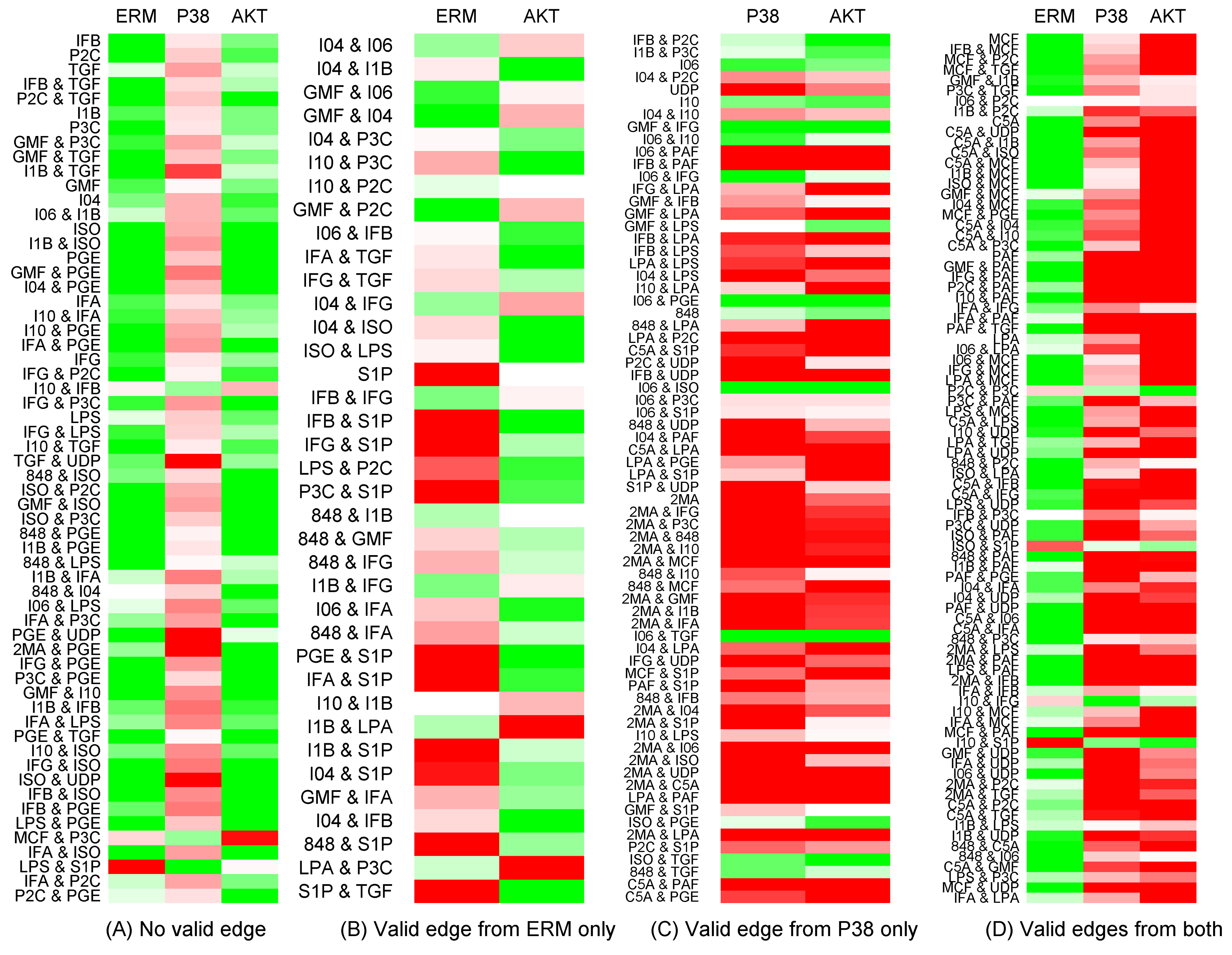

Supplement: Figure S1 — Display of experimental data corresponding to the four cases of valid paths from ERM and/or P38 to AKT in Figure 5A. (1.59 MB TIF) [file pcbi.1000654.s004.tif]

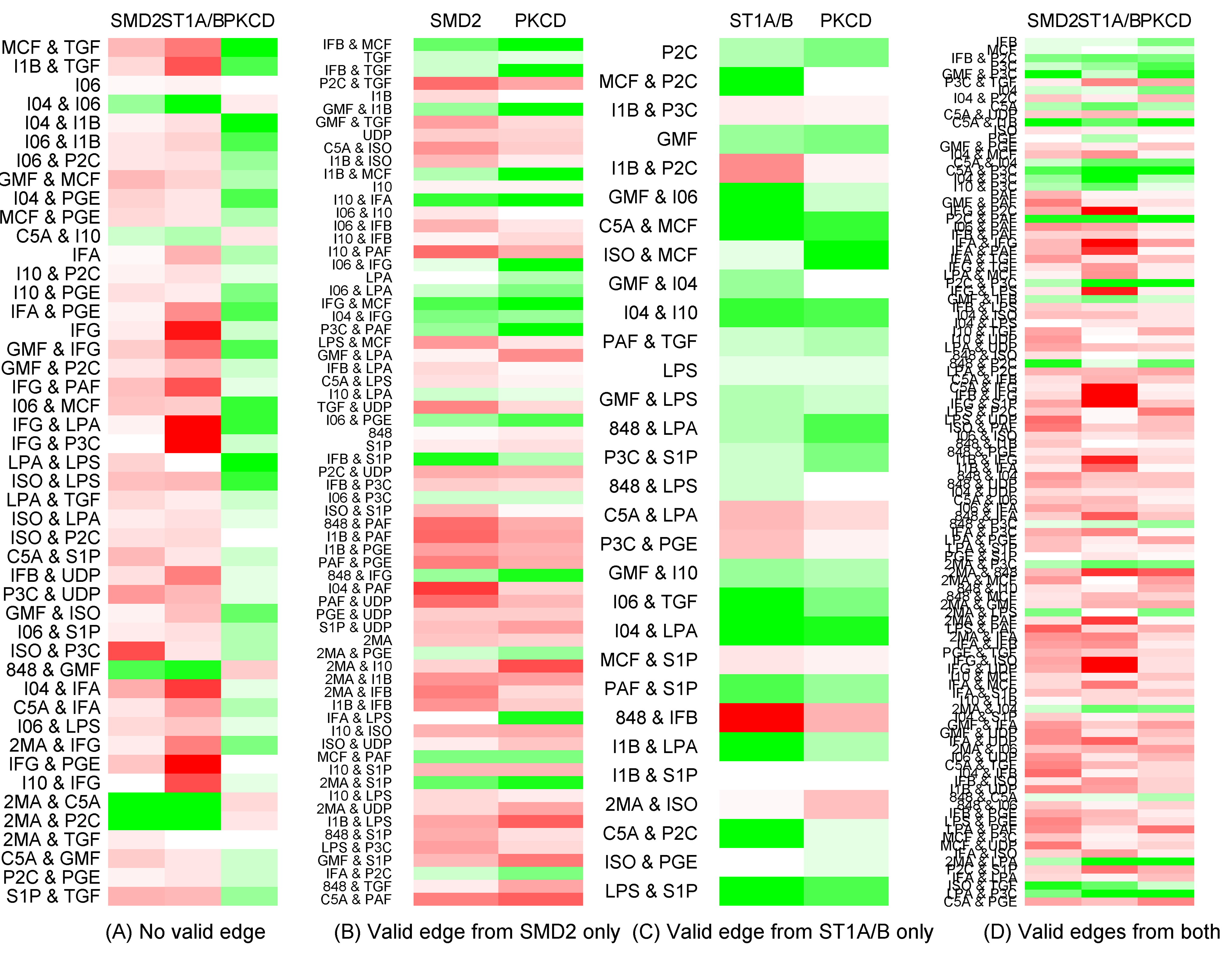

Supplement: Figure S2 — Display of experimental data corresponding to the four cases of valid paths from SMD2 (SMAD 2) and/or ST1A/B (STAT 1A/B) to PKCD in Figure 5C. (1.56 MB TIF) [file pcbi.1000654.s005.tif]

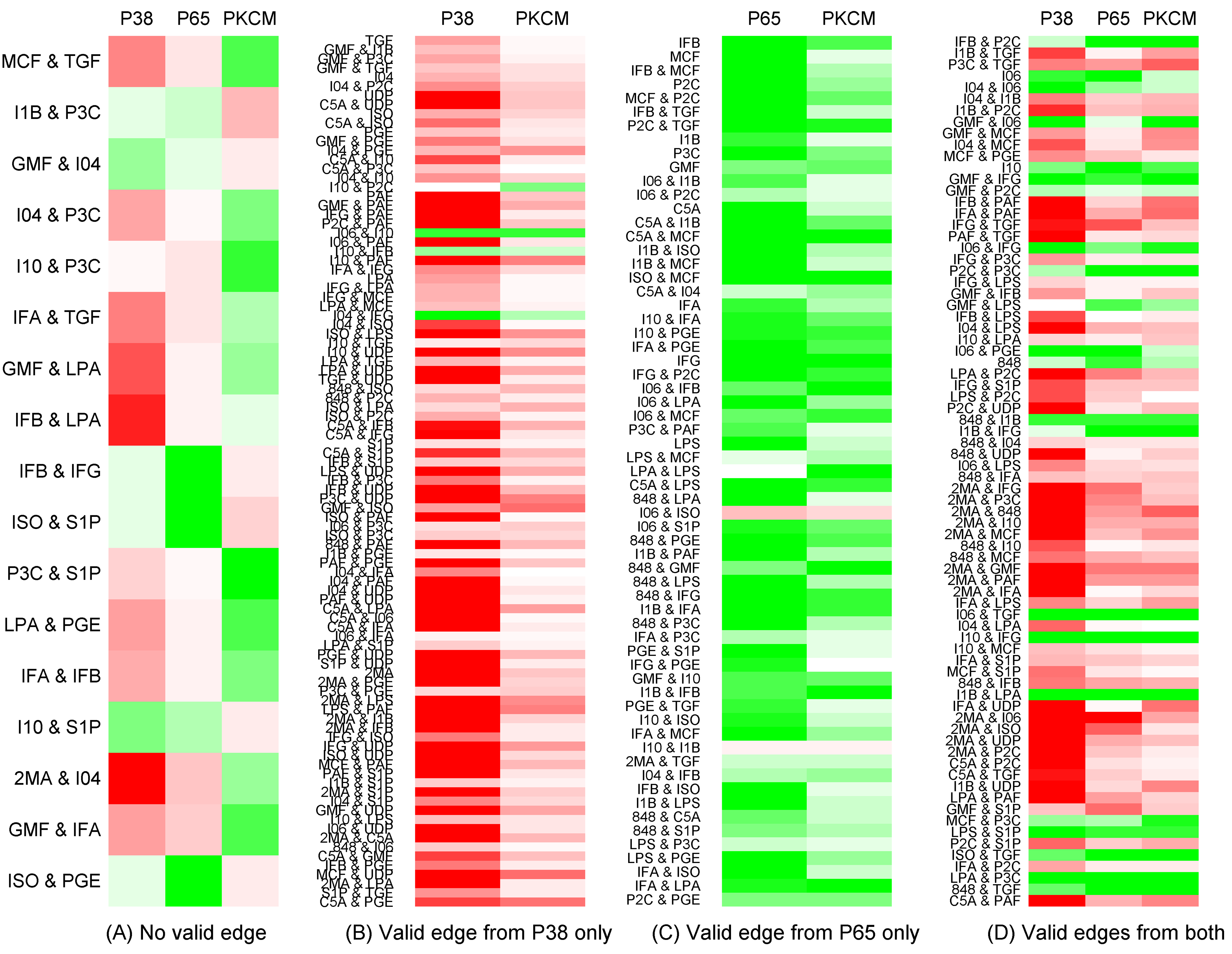

Supplement: Figure S3 — Display of experimental data corresponding to the four cases of valid paths from P38 and/or P65 to PKCM in Figure 5D. (1.51 MB TIF) [file pcbi.1000654.s006.tif]

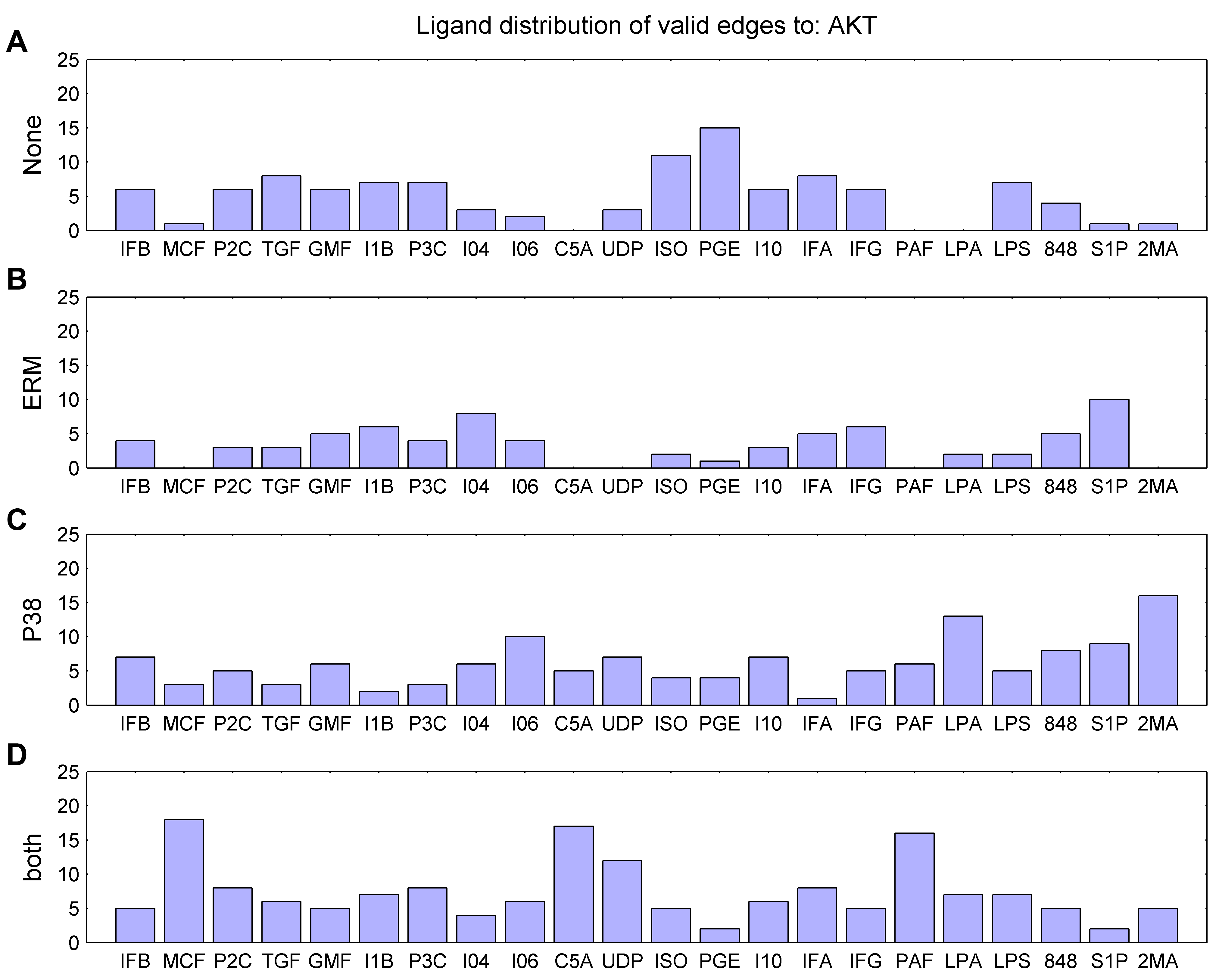

Supplement: Figure S4 — Ligands distribution for all four cases of AKT activation (displayed in Figure 5A). X-axis and Y-axis represent the name of ligand and counts of the cases respectively. For dual ligand experiment, the case is added to both of the ligands. (0.31 MB TIF) [file pcbi.1000654.s007.tif]

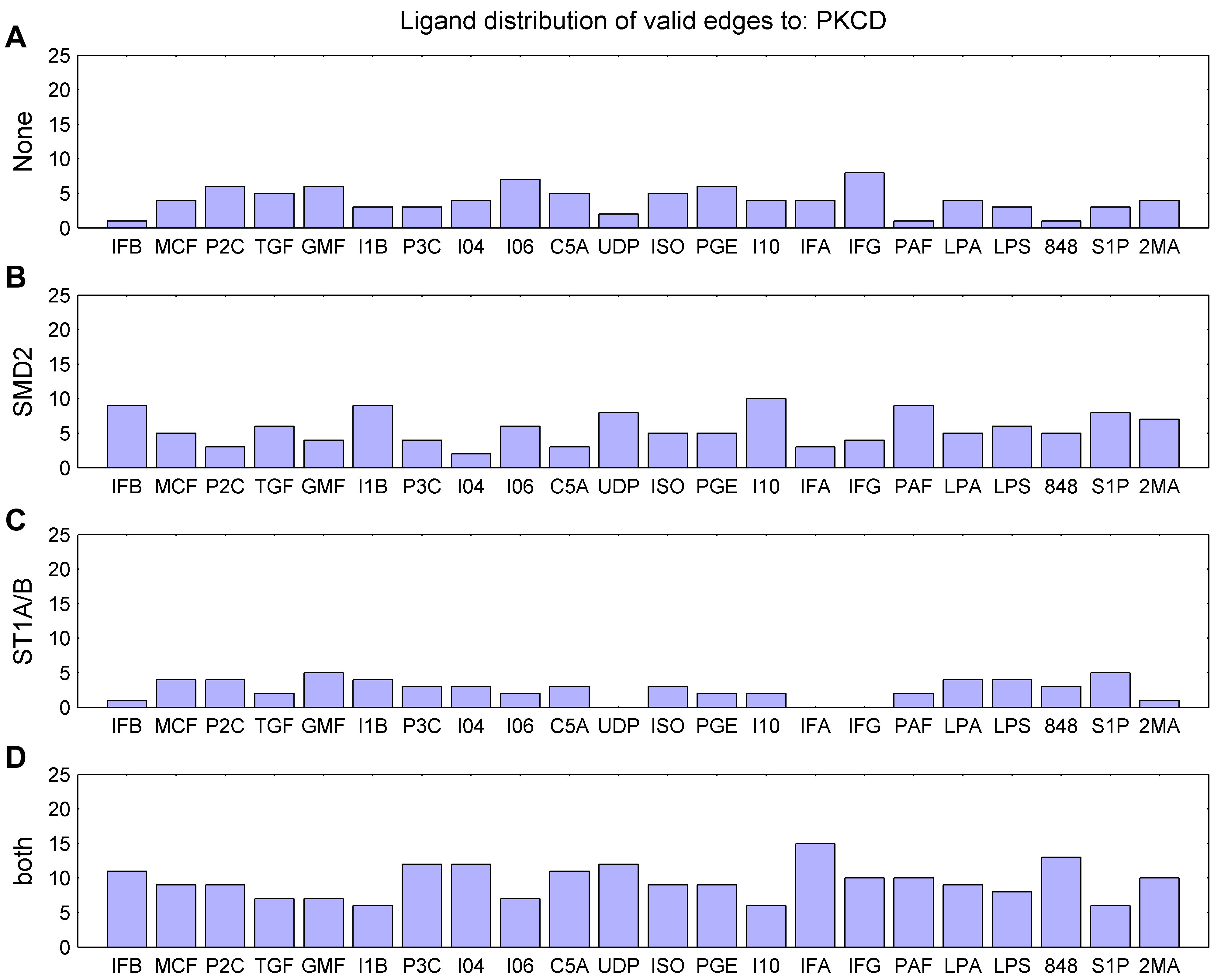

Supplement: Figure S5 — Ligands distribution for all four cases of PKCD activation (displayed in Figure 5C). X-axis and Y-axis represent the name of ligand and counts of the cases respectively. For dual ligand experiment, the case is added to both of the ligands. (0.31 MB TIF) [file pcbi.1000654.s008.tif]

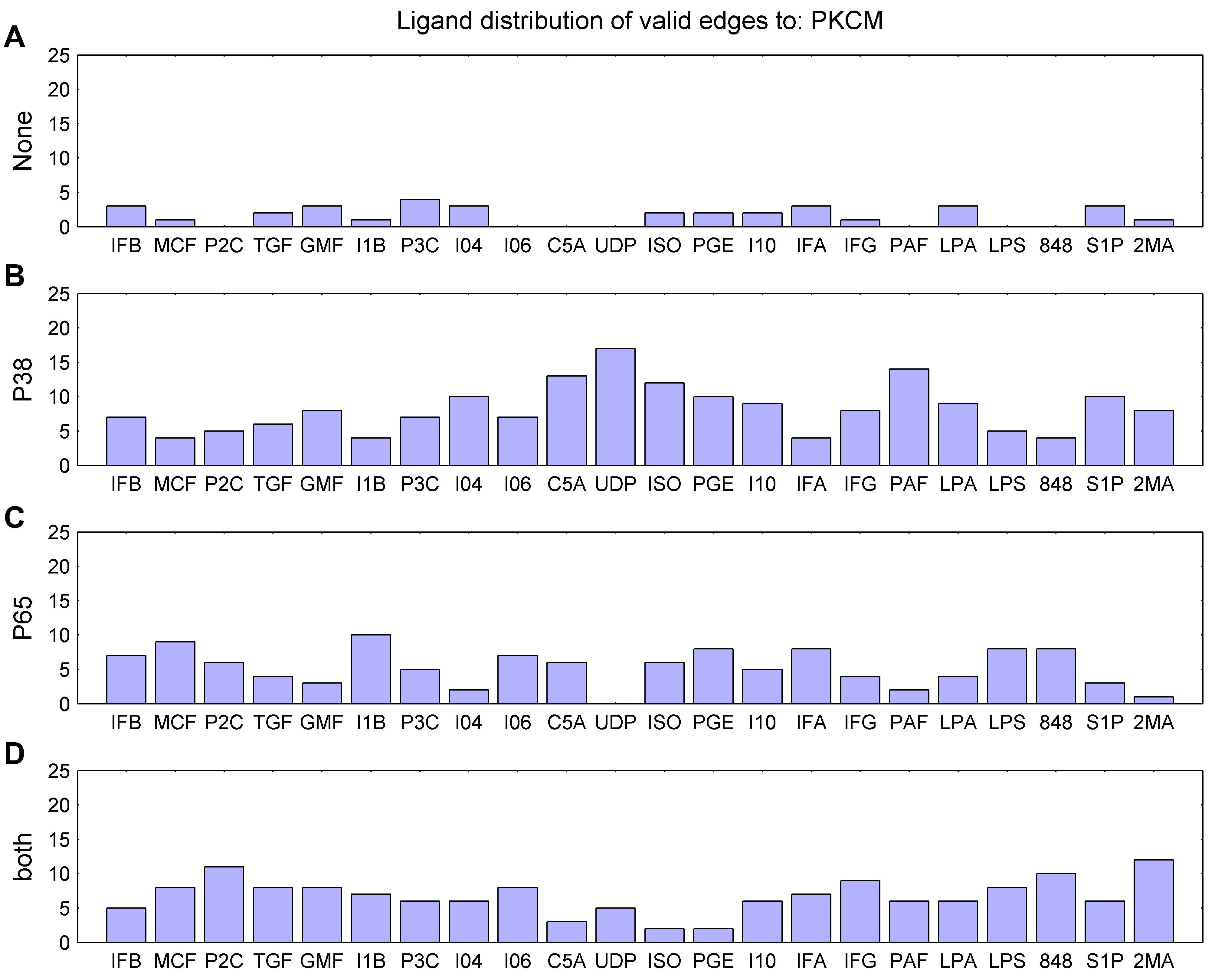

Supplement: Figure S6 — Ligands distribution for all four cases of PKCM activation (displayed in Figure 5D). X-axis and Y-axis represent the name of ligand and counts of the cases respectively. For dual ligand experiment, the case is added to both of the ligands. (0.31 MB TIF) [file pcbi.1000654.s009.tif]

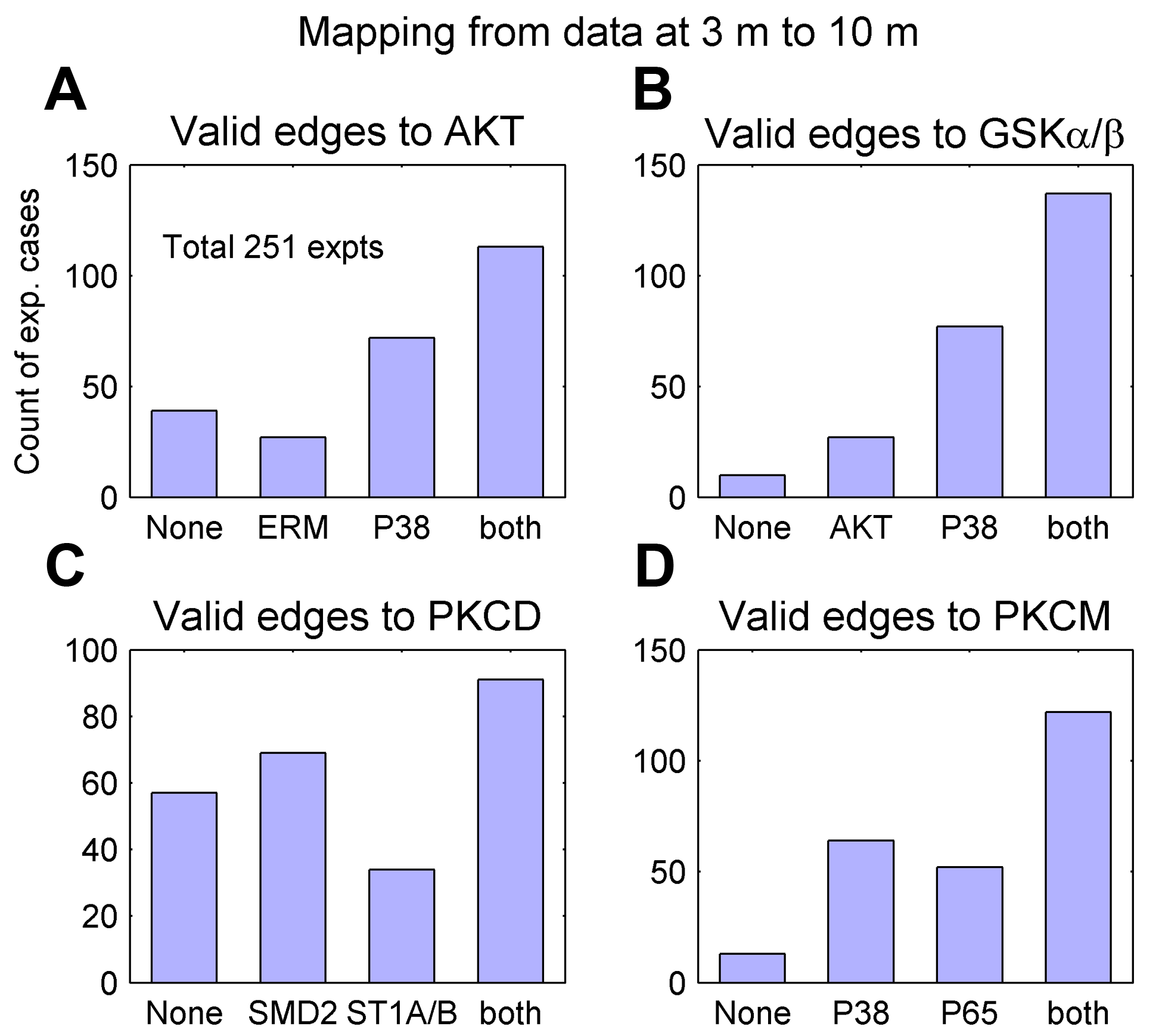

Supplement: Figure S7 — Distribution of the consistency of pathways for nodes which are activated through two different pathways (similar to Figure 5), based on the mapping of data from 3 min to 10 min. X-axis represents four possibilities: (1) neither path consistent (‘None’), (2) path 1 consistent, but not 2 (3) path 2 consistent but not 1, (4) both paths consistent. Y-axis represents the number of experiments counted for each case. (0.32 MB TIF) [file pcbi.1000654.s010.tif]

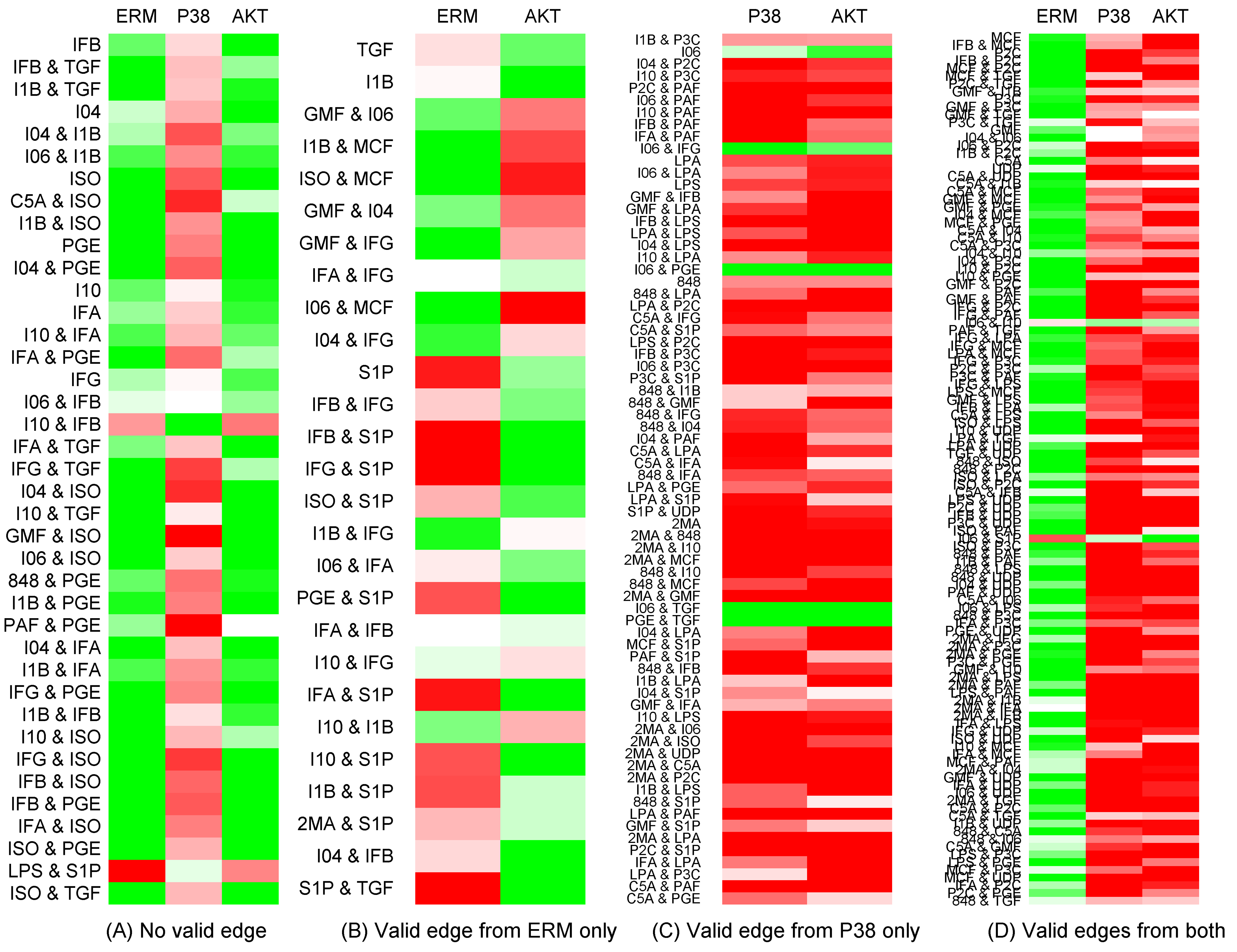

Supplement: Figure S8 — Display of experimental data corresponding to the four cases of valid paths from ERM and/or P38 to AKT in Figure S7A based on the mapping of data from 3 min to 10 min. (1.51 MB TIF) [file pcbi.1000654.s011.tif]

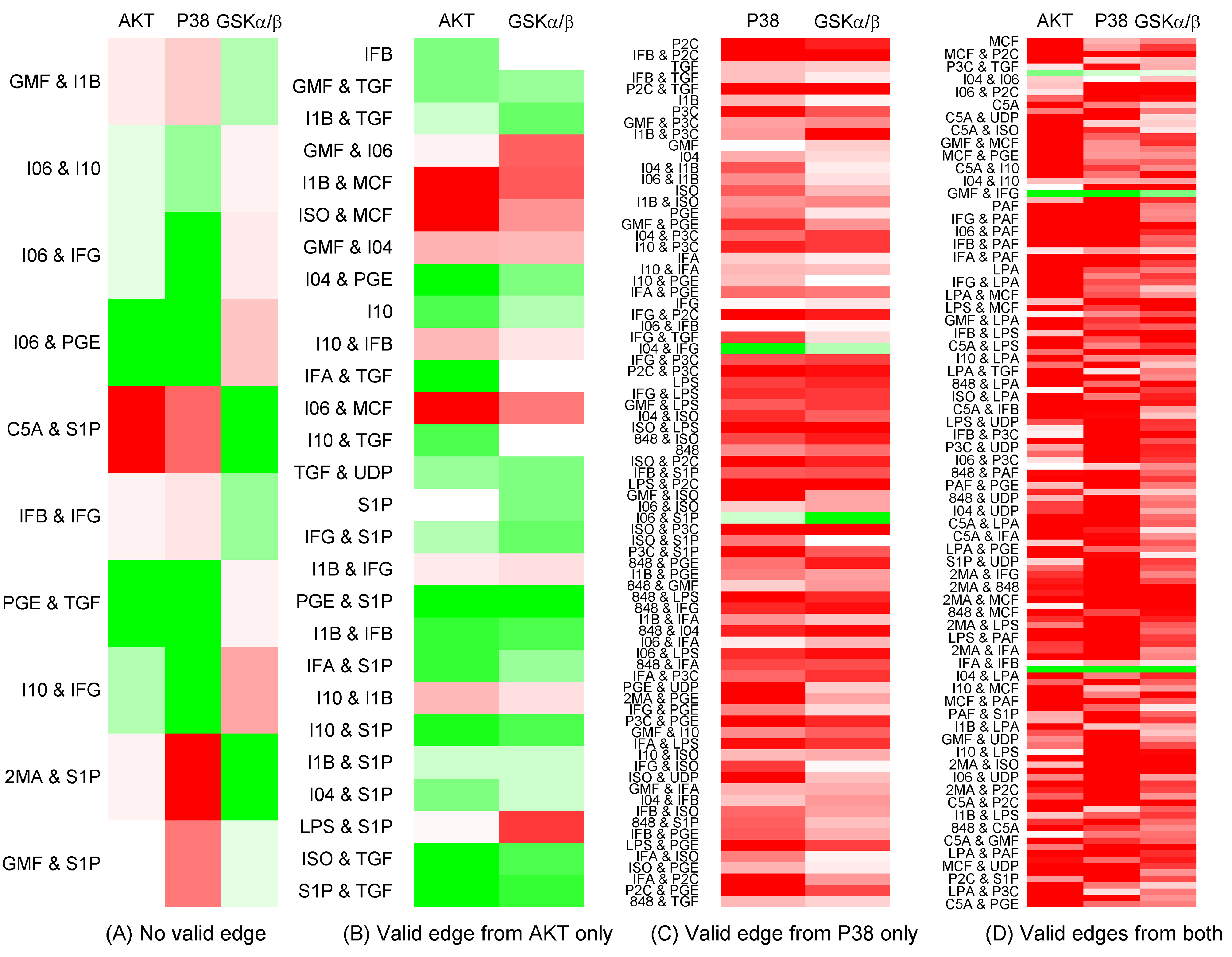

Supplement: Figure S9 — Display of experimental data corresponding to the four cases of valid paths from P38 and/or AKT to GSKα/β in Figure S7B based on the mapping of data from 3 min to 10 min. (1.26 MB TIF) [file pcbi.1000654.s012.tif]

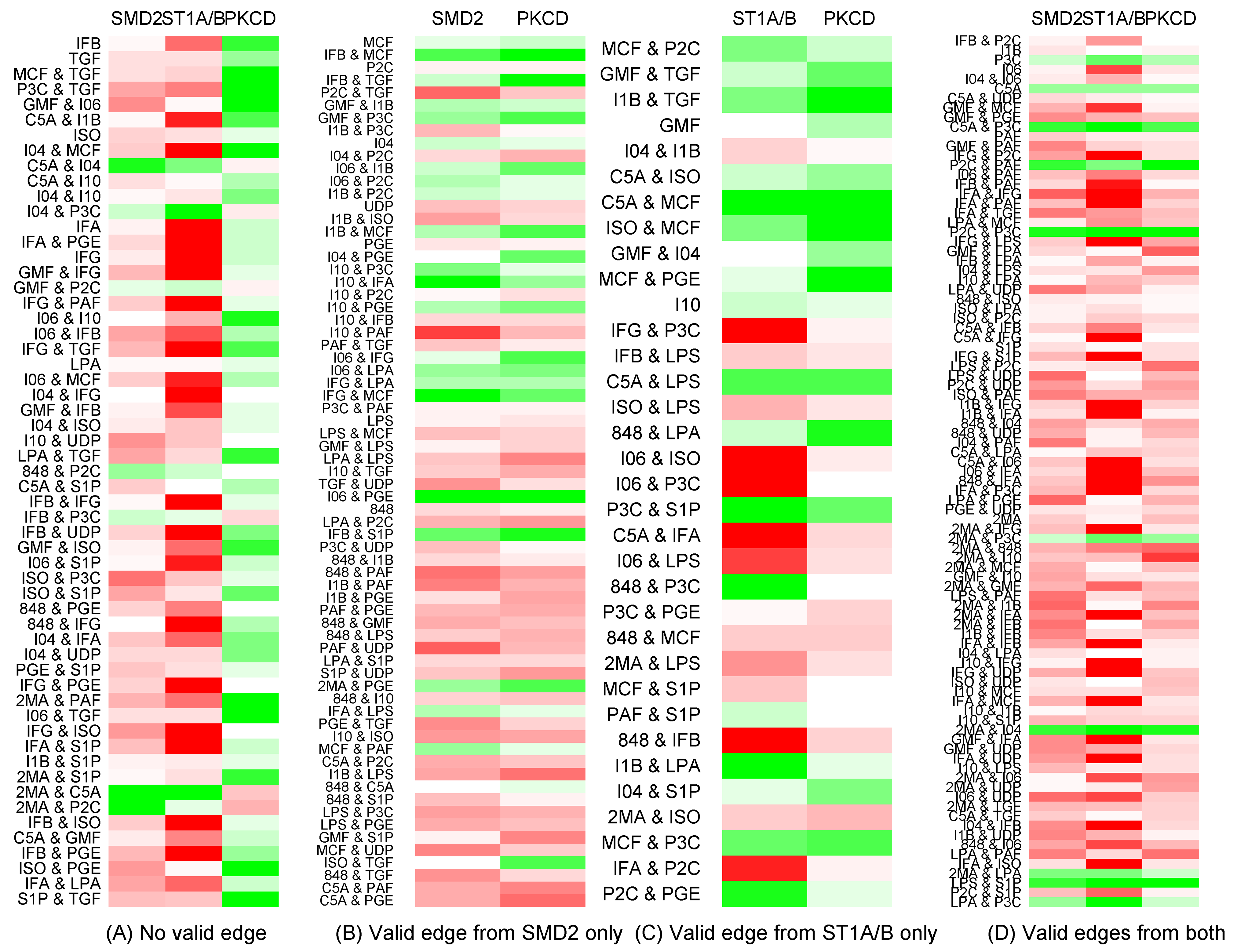

Supplement: Figure S10 — Display of experimental data corresponding to the four cases of valid paths from SMD2 (SMAD 2) and/or ST1A/B (STAT 1A/B) to PKCD in Figure S7C based on the mapping of data from 3 min to 10 min. (1.59 MB TIF) [file pcbi.1000654.s013.tif]

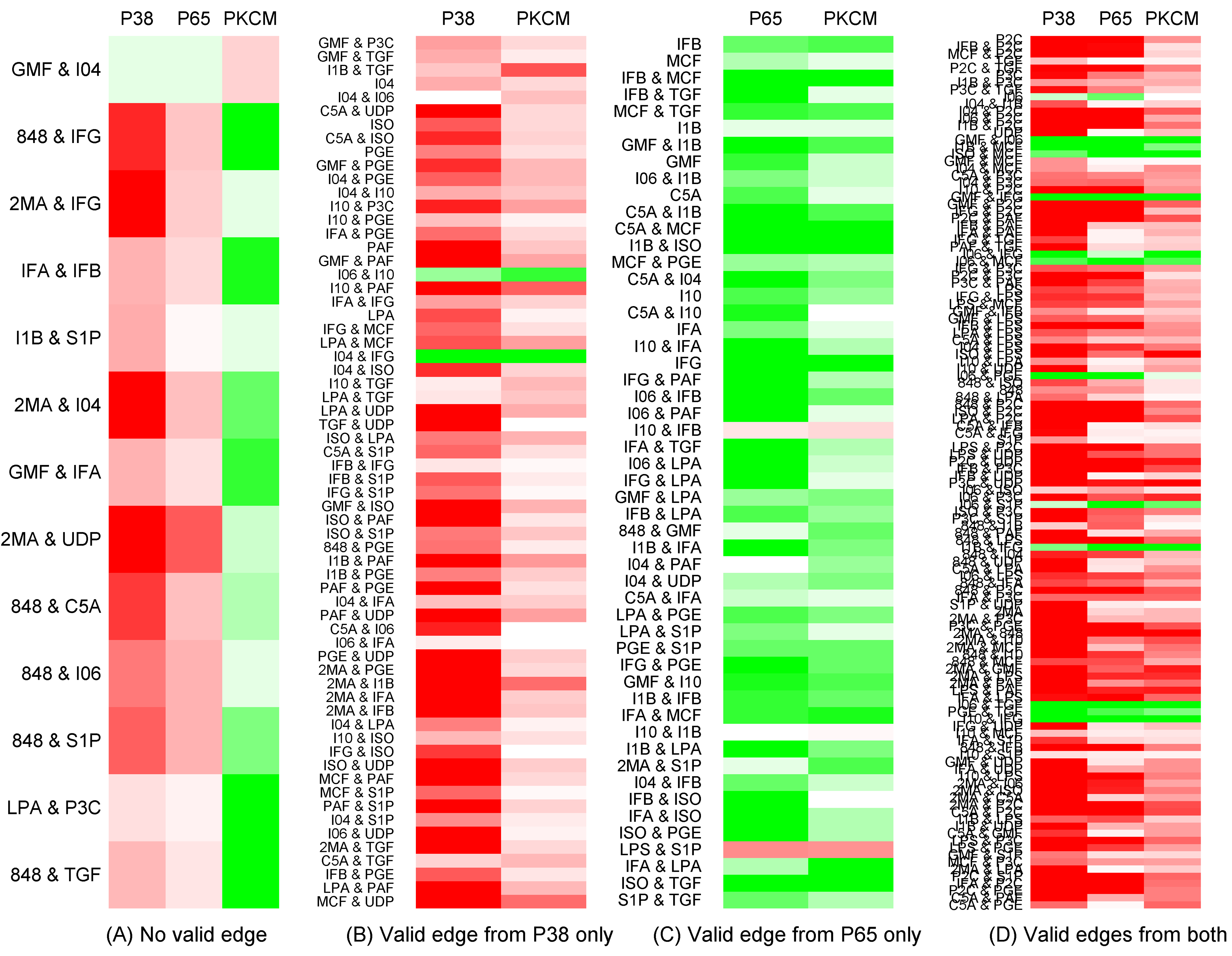

Supplement: Figure S11 — Display of experimental data corresponding to the four cases of valid paths from P38 and/or P65 to PKCM in Figure S7D based on the mapping of data from 3 min to 10 min. (1.46 MB TIF) [file pcbi.1000654.s014.tif]

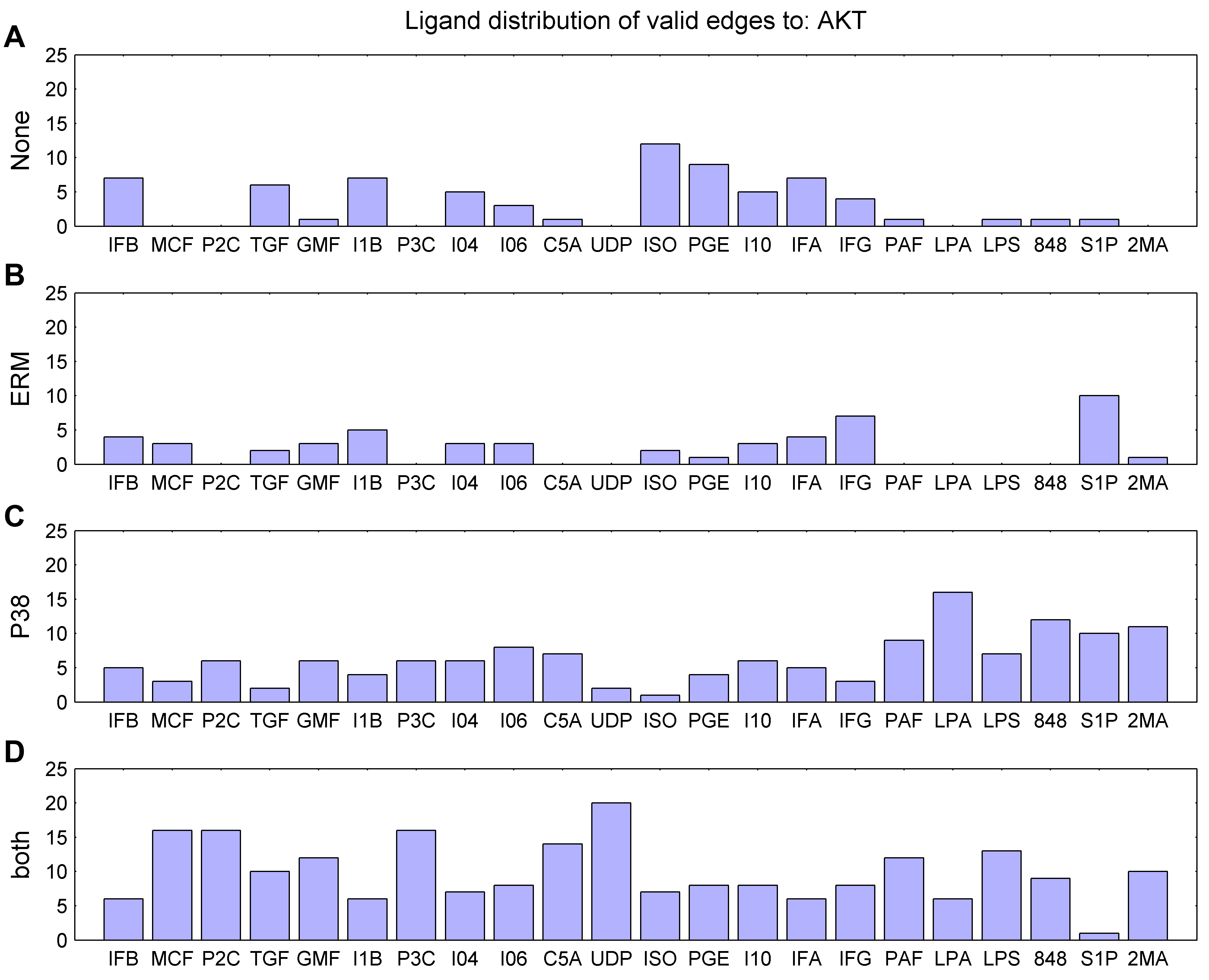

Supplement: Figure S12 — Based on the mapping of data from 3 min to 10 min: Ligands distribution for all four cases of AKT activation (summarized in Figure S7A). X-axis and Y-axis represent the name of ligand and counts of the cases, respectively. For dual ligand experiments, the case is added to both of the ligands. The panels A–D also correspond to the heat-maps of Figure S8 A–D, respectively. (0.31 MB TIF) [file pcbi.1000654.s015.tif]

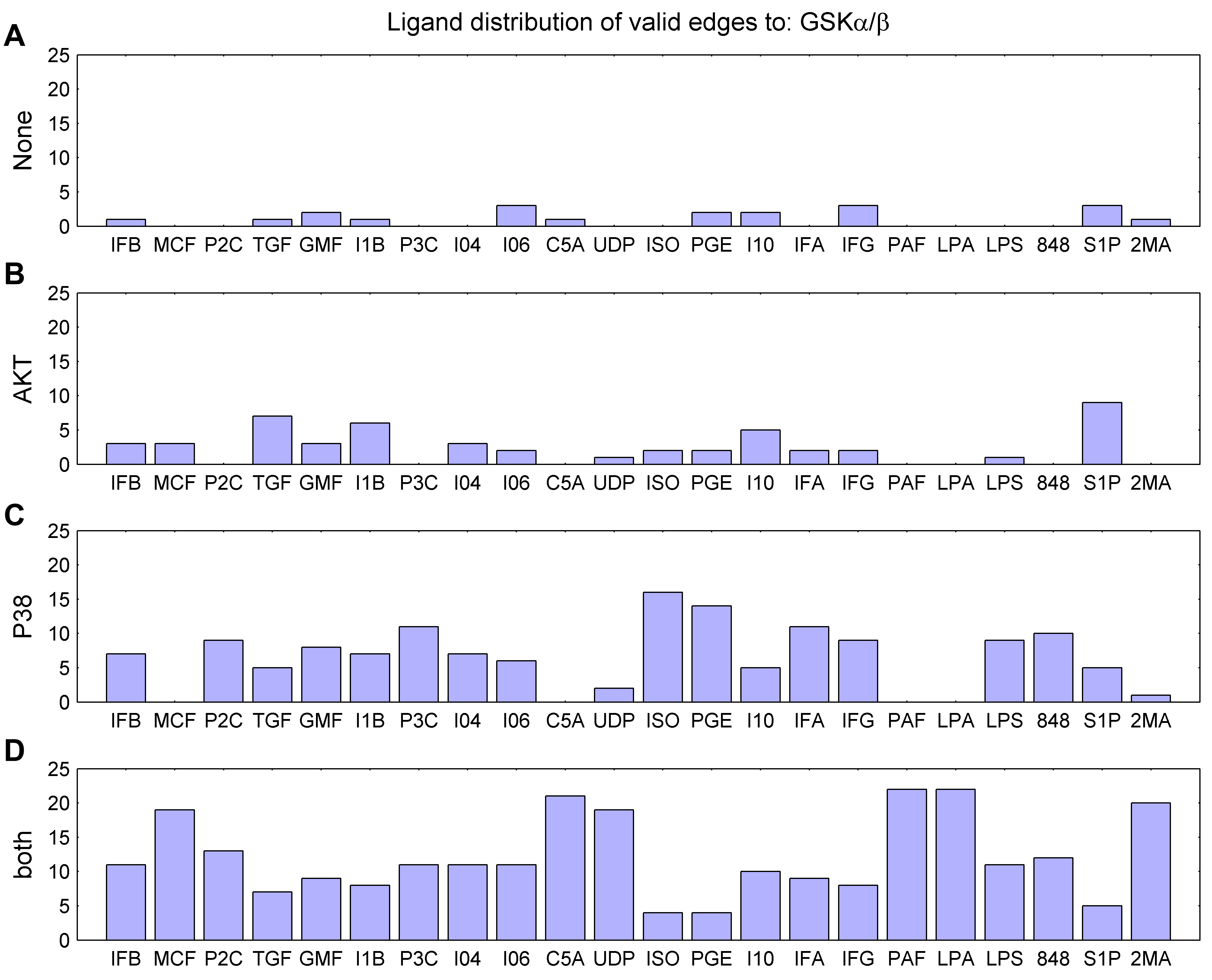

Supplement: Figure S13 — Based on the mapping of data from 3 min to 10 min: Ligand distribution for all four cases of GSKα/β activation (summarized in Figure S7B). X-axis and Y-axis represent the name of ligand and counts of the cases, respectively. For dual ligand experiments, the case is added to both of the ligands. The panels A–D also correspond to the heat-maps of Figure S9 A–D, respectively. (0.31 MB TIF) [file pcbi.1000654.s016.tif]

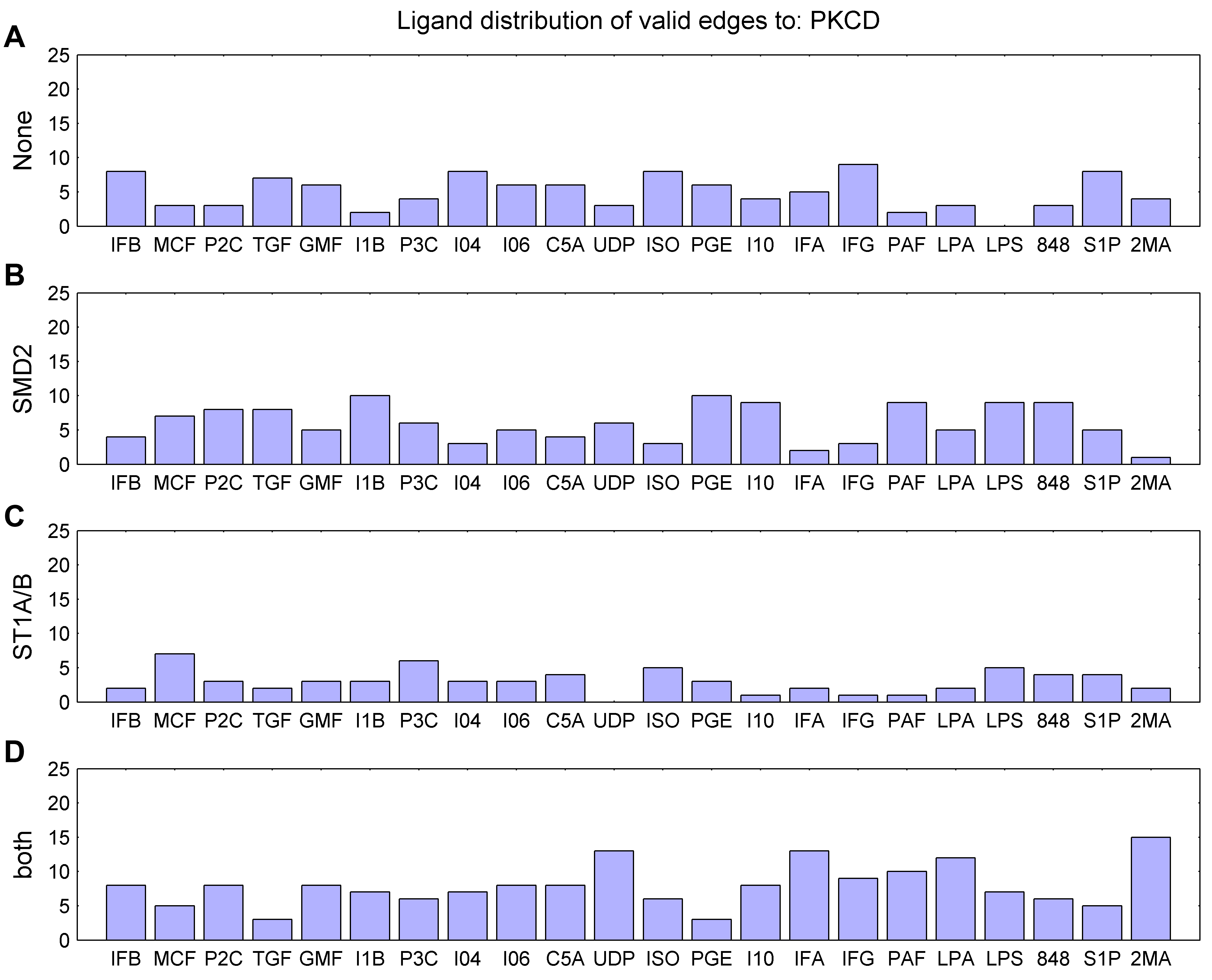

Supplement: Figure S14 — Based on the mapping of data from 3 min to 10 min: Ligand distribution for all four cases of PKCD activation (summarized in Figure S7C). X-axis and Y-axis represent the name of ligand and counts of the cases, respectively. For dual ligand experiments, the case is added to both of the ligands. The panels A–D also correspond to the heat-maps of Figure S10 A–D, respectively. (0.31 MB TIF) [file pcbi.1000654.s017.tif]

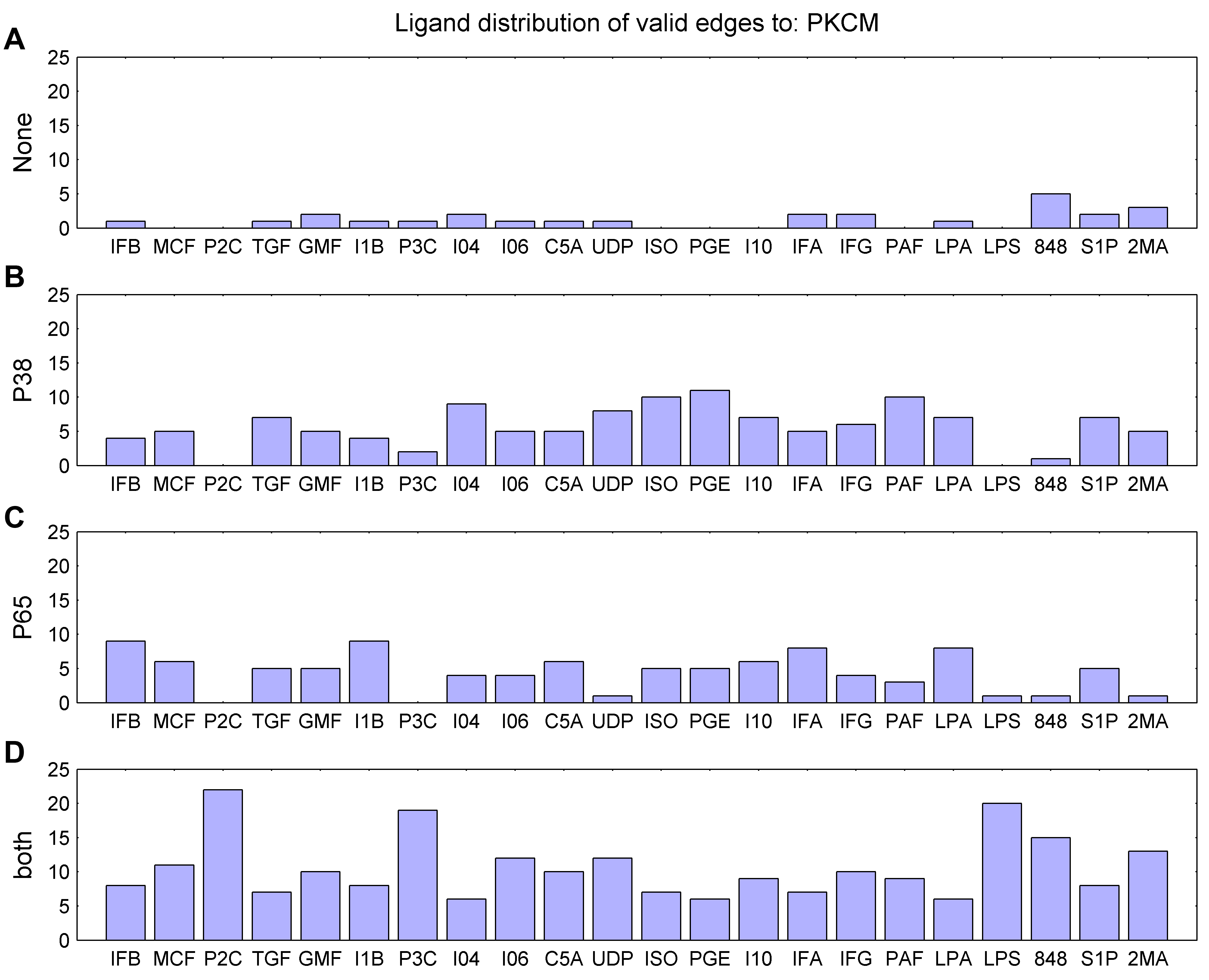

Supplement: Figure S15 — Based on the mapping of data from 3 min to 10 min: Ligand distribution for all four cases of PKCM activation (summarized in Figure S7D). X-axis and Y-axis represent the name of ligand and counts of the cases, respectively. For dual ligand experiments, the case is added to both of the ligands. The panels A–D also correspond to the heat-maps of Figure S11 A–D, respectively. (0.31 MB TIF) [file pcbi.1000654.s018.tif]
