# Supplementary material for: Identification of Crosstalk between Phosphoprotein Signaling Pathways in RAW 264.7 Macrophage Cells
Source: PLoS Comput Biol. 2010 Jan 29;6(1):e1000654. doi: 10.1371/journal.pcbi.1000654 (PMC2813256; doi:10.1371/journal.pcbi.1000654)
Supplement: Text S1 — F-test in K-fold Cross-Validation (0.08 MB PDF) [file pcbi.1000654.s019.pdf]

F-test in K-fold Cross-Validation

The analysis and procedure presented below is based upon least-squares approach. Similar arguments hold true for the application of PLS as well, although the concept of degrees of freedom (DOF) is more complicated in PLS.

Least-squares approach: Let X and Y be the input and output data (single output), respectively. Let *n* be the number of data points and p be the number of input predictors. Let the model be:

$$Y = Xb + E \tag{1}$$

Then the least squares estimate of b is $\hat{b} = (X^T X)^{-1} X^T Y$. Predicted value of Y is $\hat{Y} = X\hat{b} = X(X^T X)^{-1} X^T Y$ and the residual vector is $E = Y - \hat{Y} = [I - X(X^T X)^{-1} X^T]Y$. *Iff* mean(*Y*) = 0 *then* mean(*E*) = *0*. This constraint reduces the DOF of $\sum_i e_i^2$ by 1. Further, we have, $X^T E = X^T (Y - \hat{Y}) = X^T [I - X(X^T X)^{-1} X^T]Y = 0$ regardless of whether Y is mean-centered or not. These *p* constraints further reduce the DOF of $\sum_i e_i^2$ by *p*. Thus, effective DOF of $\sum_i e_i^2$ is (*n – p - 1*).

In the application of K-fold CV, let *n* be the number of total data points and *f* be the number of folds (*f* = 10 here) so that each fold contains *m = n/f* data points for the test set and $m \times (f-1)$ data points for the training set. Let p be the number of input predictors. Consider one output variable at a time. Let $r_{i,j}$ be the residual for the jth sample in ith fold in training set. Then, $S_i = \sum_j r_{i,j}^2 \sim \chi^2_{m(f-1)-p-1} \ (\forall i)$ is true. However, the distribution of $S = \sum_i S_i = (\sum_i \sum_j r_{i,j}^2)$ is not clear because, out of all the $f \times m \times (f-1) = n \times (f-1)$ samples in all the training sets of different folds, only *n* samples are truly independent.

In our F-test, we are computing the F-statistic as follows:

For the test sets:

$$S_{test,i} = \sum_j r_{test,i,j}^2; \quad DOF(S_{test,i}) = m; \quad S_{test} = \sum_i S_{test,i} = \sum_i \sum_j r_{test,i,j}^2; \quad DOF(S_{test}) = m * f \tag{2}$$

For the training sets:

$$S_{train,i} = \sum_j r_{train,i,j}^2; \quad DOF(S_{train,i}) = m \times (f-1) - p - 1; \quad S_{train} = \sum_i S_{train,i} = \sum_i \sum_j r_{train,i,j}^2 \tag{3}$$

$m \times (f-1) \times f$ numbers of residual terms are included in S_{train} in which $m \times f$ independent residual terms are repeated $(f-1)$ times (approximately). Hence, the statistic $S_{train}/(f-1)$ contains $m \times f$ independent residual terms and follows chi-square distribution with $DOF(S_{train}/(f-1)) \approx m \times f$. Thus, the statistic F follows,

$$F = \frac{S_{test}/(m \times f)}{\left(S_{train}/(f-1)\right)/(m \times f)} = \frac{S_{test}/(m \times f)}{S_{train}/(m \times (f-1) \times f)} \tag{4}$$

The denominator in the above expression does not include any effect of the number of input variables p. The effect of p can be achieved by considering the statistic

$$F = \frac{S_{test}/(m \times f)}{S_{train}/((m \times (f-1) - p - 1) \times f)} \tag{5}$$

So, the equivalent DOF is, $DOF_{train} = (m \times (f-1) - p - 1) \times f/(f-1)$. Hence, $F \sim F(n, DOF_{train})$ and, we have tested the hypothesis for the significance level, α:

$$\begin{aligned} H_0 &: F < F_\alpha(n, DOF_{train}) \\ H_1 &: F > F_\alpha(n, DOF_{train}) \end{aligned} \tag{6}$$

where $F_\alpha(n_1, n_2)$ denotes inverse cumulative F-distribution value for DOF n_1 and n_2 at the significance level of α.
